# Supplementary material for: Site-specific electrodeposition enables self-terminating growth of atomically dispersed metal catalysts
Source: Nat Commun. 2020 Sep 11;11:4558. doi: 10.1038/s41467-020-18430-8 (PMC7486907; doi:10.1038/s41467-020-18430-8)
Supplement: Supplementary file 1 — Supplementary Information [file 41467_2020_18430_MOESM1_ESM.pdf]

Supplementary Information

Site-specific electrodeposition enables self-terminating growth of atomically dispersed metal catalysts

Shi et al.

Supplementary Methods

Synthesis of chemically exfoliated transition metal dichalcogenides (TMDs)

For ce-MoS_2 nanosheets, pristine bulk MoS_2 powder (0.6 g) was mixed with *n*-butyllithium in hexane (2.5 M, 5 mL) at 60 °C under an argon atmosphere. After 48 h, the resulting suspension was subjected to centrifugation (5000 rpm, 10 min, 25°C) and the solid was washed with hexane (3 × 50 mL). After the lithium intercalation step, the resulting black compound was immediately transferred into water and ultrasonicated for 1 h. The exfoliated suspension was further dialyzed for 7 days (BIOSHARP dialysis membrane, USA, molecular weight cut-off 14 kD). The suspension was then subjected to centrifugation (8000 rpm, 15 min, 25°C) to remove non-exfoliated material. Approximately 100 mL of supernatant was collected and freshly used for characterization and electrochemical studies. For the synthesis of ce-MoS_2 (MoS_2 in excess), pristine bulk MoS_2 powder (0.6 g) was mixed with *n*-butyllithium in hexane (2.5 M, 0.5 mL) with the addition of hexane (4.5 mL) at 60 °C under an argon atmosphere. For the synthesis of ce-WS_2 and ce-$MoSe_2$, pristine WS_2 and $MoSe_2$ powder with an equimolar amount of MoS_2 powder were added, respectively. The synthesis then followed the same procedure as that for ce-MoS_2. All our experiments and measurements were carried out within 3 days to avoid aging effects.

Synthesis of Pt single atoms on different substrates

Pt-SAs/WS_2 and Pt-SAs/$MoSe_2$ were prepared according to similar procedures as Pt-SAs/MoS_2, instead using ce-WS_2 and ce-$MoSe_2$ as the supporting substrates, respectively. The resulting product was transferred into an argon-filled glovebox for storage at room temperature.

Synthesis of Pd-SAs/MoS_2 and Rh-SAs/MoS_2

Pd-SAs/MoS_2 and Rh-SAs/MoS_2 were also prepared according to similar procedures as Pt-SAs/MoS_2, except for using 5 mM K_2PdCl_4 and $RhCl_3$ as the metal precursors for galvanic displacement. The resulting product was transferred into an argon-filled glovebox for storage at room temperature.

Synthesis of Sn-SAs/MoS_2, Bi-SAs/MoS_2, and Pb-SAs/MoS_2

The M-SAs/MoS_2 catalysts (M=Sn, Bi, and Pb) were prepared according to similar procedures using the corresponding metal salt precursors (SnO, $Bi(NO_3)_3$, and $Pb(NO_3)_2$) and various deposition potentials. The deposition potentials are determined according to the cyclic

voltammograms (CVs; Supplementary Fig. 9), at which only the UPD process occurs. The resulting product was transferred into an argon-filled glovebox for storage at room temperature.

Synthesis of I_2-oxidized ce-MoS_2

Ce-MoS_2 nanosheets (15 mg) were treated with 0.15 M iodine solution in acetonitrile (15 mL). After 8 days of continuous stirring at room temperature, the resulting product was washed sequentially with acetonitrile (3 × 40 mL), 2-propanol (3 × 40 mL), ethanol (3 × 40 mL), and water (3 × 40 mL).

Synthesis of Pt-nanoparticle-modified ce-MoS_2 nanosheets (Pt-NPs/MoS_2)

Ce-MoS_2 nanosheets (20 mg), K_2PtCl_4, (10.6 mg), PVP (150 mg), and water (15 mL) were mixed and then stirred at room temperature for 30 min. Next, a solution of $NaBH_4$ (200 mg) in ice-cold deionized (DI) water (10 mL) was rapidly injected into the suspension. After stirring for another 30 min, the resulting product was collected by centrifugation (5000 rpm, 10 min, 25°C) and washed three times with water. Inductively coupled plasma optical emission spectrometry (ICP-OES) analysis showed that the loading of Pt in Pt-NPs/MoS_2 was approximately 17 wt%.

Large-scale synthesis of Pt-SAs/MoS_2

Typically, single-atom catalysts synthesized in the laboratory significantly outperform commercial catalysts. However, most of these nanoscale studies are fundamental, and the technology is not scaled up for widespread adoption. Therefore, we designed a site-specific electrodeposition (SSED) device for potential macroscale production. The electrolysis system was constructed from a "U-type" electrochemical cell with a proton-exchange membrane (Supplementary Fig. 11). In this three-electrode configuration, a graphite rod and Ag/AgCl (saturated KCl) were used as the working and reference electrodes, respectively, in one half-reaction electrochemical cell with an electrolyte of argon-saturated 2 mM $CuSO_4$ (20 mL) containing 0.1 M H_2SO_4 and ce-MoS_2 (300 mg). A Pt wire was used as the counter electrode in the other half-reaction electrochemical cell with an electrolyte of 0.1 M H_2SO_4 (20 mL). A constant potential of 0.1 V was applied at the working electrode for 10 min under continuous stirring, during which a fluffy black powder was gradually adsorbed onto the graphite rod. Then, a solution of 0.05 M H_2SO_4 containing 5 mM K_2PtCl_4 (5 mL; degassed with bubbling argon for 30 min) was immediately injected into the cell containing the black powder, and the reaction was allowed to stir for 30 min. The resulting product was washed several times with

water, frozen by liquid nitrogen, and lyophilized overnight. All the synthetic procedures were conducted at ambient temperature. The resulting product was then transferred into an argon-filled glovebox for storage.

Operando Raman Spectroscopy of ce-MoS_2 during the UPD of Cu

An in-house-built electrochemical cell was used for the operando Raman spectroscopy experiments (Supplementary Fig. 17). In a typical experiment, an indium tin oxide (ITO) substrate spin-coated (acceleration rate: 1000 rpm; rotation speed: 3000 rpm) with ce-MoS_2 nanosheets was utilized as the working electrode. A polydimethylsiloxane (PDMS) membrane, which was synthesized according to standard procedures, was used to control the exposed area of the working electrode. A platinum wire and a Ag/AgCl electrode served as the counter and reference electrodes, respectively. A potentiostat was used to apply potentials of +0.10 V to the working electrode while the Raman spectra were collected. A confocal Raman microscope (FTRaman Spectrometer, Bruker) was used to acquire *in situ* and operando Raman spectra using a 532 nm laser with an acquisition time of 15 s for each spectrum. During the reaction, electrolyte (2 mM $CuSO_4$ containing 0.1 M H_2SO_4, approx. 200 μL) was added. A CHI 660E potentiostat was used to establish the electrolysis conditions for the Cu UPD.

Supplementary Notes

Supplementary Note 1. Conceiving a new electrochemical approach for ADMC preparation

Conductive substrates with high surface areas (e.g., graphene and carbon nanotubes) have often been used to electrodeposit metal nanoparticles or metallic thin films (Supplementary Fig. 1). However, this process typically leads to the formation of a multilayer bulk phase. Underpotential deposition (UPD) is an electrochemical phenomenon by which typically a metal cation (e.g., Cu^{2+}, Ag^{+}, Bi^{3+}, and Pb^{2+}) is deposited onto a solid metal (for example, Au, Pd and Pt) at a potential more positive than its equilibrium potential (the potential at which it deposits onto itself)[1]. This phenomenon usually arises from the strong interaction between the depositing metal and the substrate: the metal–substrate interaction is energetically favorable compared to the metal–metal interaction in the crystal lattice of the bare metal. UPD can then be understood to be when a metal can deposit onto another material more easily than it can deposit onto itself[2]. Adzic *et al.* have reported various single-layer core–shell nanostructures through underpotentially depositing a foreign metal (e.g., Cu) onto a naked metal support (e.g., Au, Pd, and Pt), followed with metal exchange by the desired Pt-group metal[3-6]. However, single-layer Au@Pt core-shell structures are almost completely and rapidly deactivated because of the ease of surface rearrangements between the Pt islands and exposed Au substrate at the electrode–electrolyte interface. Inspired by these findings, we propose that the synthesis of ADMCs might be realized through SSED on a supporting substrate with isolated active sites for the UPD.

Supplementary Note 2. Estimation of Cu coverage

According to the calculation shown below, we can estimate the coverage of Cu atoms on the ce-MoS_2 nanosheets (θ_{Cu}). The amount of Cu can be obtained by integration of the cathodic peak corresponding to Cu UPD (Supplementary Fig. 4b).

$$n_{\mathrm{Cu}} = \frac{Q_{\mathrm{Cu}}}{96500} \times \frac{1}{2} = \frac{4.162\times10^{-5}}{96500\times2} = 2.156 \times 10^{-10}\ \mathrm{mol} \quad (1)$$

The value of 96500 is the Faraday constant in C mol^{-1}. The factor 1/2 reflects that two electrons are required to reduce one Cu^{2+} ion to Cu ($Cu^{2+} + 2e^- \rightarrow Cu$).

The amount of the ce-MoS_2 nanosheets on GCE:

$$m_{MoS_2} = 1\ \mathrm{mg\ ml^{-1}} \times \frac{1}{8} \times 5\ \mu\mathrm{L} = 6.25 \times 10^{-4}\ \mathrm{mg} \quad (2)$$

$$n_{MoS_2} = \frac{6.25\times10^{-4}\ mg}{160\ g/mol} = 3.9 \times 10^{-9}\ mol \quad (3)$$

The coverage of Cu atoms on the ce-MoS_2 nanosheets is thus:

$$\theta_{Cu} = \frac{2.156\times10^{-10}\ \mathrm{mol}}{3.9\times10^{-9}\ mol\times2} = 2.76\% \quad (4)$$

The factor 1/2 reflects that one mole of MoS_2 contains two moles of S atoms.

According to our experimental characterizations and DFT simulations, Pt atom is attached on the Mo top site through three Pt—S coordinate bond. The theoretical coverage of Cu on MoS_2 should be 33.3%. However, owing to the existence of many S defects and localized structural distortion, the real coverage of Cu atoms (2.76%) on the ce-MoS_2 nanosheets is far less than the theoretical one (33.3%), which indirectly confirms that SSED Cu on ce-MoS_2 is truly less than a monolayer.

Supplementary Note 3. Pt SA loading amount

Owing to a local one-to-one galvanic exchange of Cu atoms, we can theoretically estimate the amount of Pt loaded onto Pt SA (n_{Pt}). This estimation is based on the stoichiometric conversion from Cu to Pt on the ce-MoS_2 nanosheets.

Assuming that no ce-MoS_2 nanosheets detach from the GCE and that the Faradic efficiency and the conversion efficiency from Cu to Pt are 100%, then:

$$n_{\mathrm{Pt}} = n_{\mathrm{Cu}} = \frac{Q_{\mathrm{Cu}}}{96500} \times \frac{1}{2} = \frac{4.162\times10^{-5}}{96500\times2} = 2.156 \times 10^{-10}\ \mathrm{mol} \quad (5)$$

The molar amount of Pt is equal to that of Cu (Cu_{upd} + Pt^{2+} → Cu^{2+} + Pt).

The theoretical loading amount of Pt on the ce-MoS_2 nanosheets is thus:

$$\eta = \frac{n_{\mathrm{Pt}}\times M_{\mathrm{Pt}}}{6.25\times10^{-4}} = \frac{2.156\times10^{-10}\times195}{6.25\times10^{-4}} \times 100\% = 6.73\ \mathrm{wt\%} \quad (6)$$

The experimental loading amount of Pt in Pt-SAs/MoS_2 (approximately 5.1 wt% analyzed by ICP-OES), though slightly lower than the nominal loading (approximately 6.73 wt%), has been reasonably considered as a first approximation to the theoretical amount. The small discrepancy can be ascribed to many complicated experimental factors, such as metal loss during the preparation process, Faradic efficiency/conversion efficiency less than 100%, etc..

Supplementary Note 4. Deep understanding of the site-specific UPD process on TMD materials

We obtained cyclic voltammograms (CVs) of the process of UPD of Cu adatoms on the TMDs (MoS_2, $MoSe_2$, WS_2 and WSe_2), and this process is reversible and surface-dominate (Supplementary Fig. 13). In principle, the anodic and cathodic currents will become nearly symmetric about the potential axis in the CVs if the sweep rate of the potential is sufficiently slow (Supplementary Fig. 14). The shapes, positions, and number of the UPD peaks largely depend on the substrate and the property of the electrolyte. On a single-crystal metal electrode, a very dense monolayered metal film can be formed *via* underpotential deposition. Such atom-by-atom metal film structure leads to the strong mutual interaction between metal atoms. In that case, the energy level of each deposited metal tends to be equal, and thus a very sharp current peak can be usually achieved. In our system, the underpotentially deposited single-atom metals are totally isolated and show no interactions with each other. Owing to the existence of nonuniformly distributed defects and localized structural distortion on substrate, energy level of each single-atom metal becomes a little distinctive, leading to the CV broadening (Supplementary Fig. 13).

These CVs have a pronounced current maximum at a distinct potential U_p. This means that the majority of adsorbed atoms are deposited at this potential with the least variation of adsorption energy as a function of coverage. This current peak therefore seems most suitable for characterizing the properties of the adatoms on different substrates[7].

Since electrochemical measurements show only the relative energy values, we can relate the free energy of deposition onto the supporting substrate to the free energy of deposition onto the bulk crystal[8]. The chemical potentials (μ) of bulk metal ($\mathrm{M_{bulk}}$) and supported single-atom metal ($\mathrm{M_{SA}}$) can be obtained from the equilibrium conditions for reactions:

$$\mathrm{M_{solv}^{z+}} + z\mathrm{e_{bulk}^{-}} \rightleftarrows \mathrm{M_{bulk}} \qquad (7)$$

with

$$\tilde{\mu}_{\mathrm{M_{solv}^{z+}}} + \tilde{\mu}_{\mathrm{e_{bulk}^{-}}} = \mu_{\mathrm{bulk}} \qquad (8)$$

and

$$\mathrm{M_{solv}^{z+}} + z\mathrm{e_{SA}^{-}} \rightleftarrows \mathrm{M_{SA}} \qquad (9)$$

with

$$\tilde{\mu}_{\mathrm{M_{solv}^{z+}}} + \tilde{\mu}_{\mathrm{e_{SA}^{-}}} = \mu_{\mathrm{SA}} \qquad (10)$$

where electrochemical potential, $\tilde{\mu} = \mu + ze_0\varphi$.

Considering the equal activity of the metal ions in solution (equal electrolyte composition, equal $\tilde{\mu}_{\mathrm{M}^{z+}_{\mathrm{solv}}}$), the difference in electron energies at the respective electrode potentials ($\Delta U_{\mathrm{p}} = U_{\mathrm{p}} - U_0$) arises from the difference in the free energy of bulk metal atoms (M) and the single metal atoms (S) adsorbed on a supporting substrate:

$$\Delta\mu_{\mathrm{S/M}} = \mu_{\mathrm{SA}} - \mu_{\mathrm{bulk}} = ze_0\left(U_{\mathrm{p}} - U_0\right) = ze_0\Delta U_{\mathrm{p}} \qquad (11)$$

where μ is the chemical potential in atomic units, e_0 is the electronic charge, U_0 is the equilibrium potential of the electrode $\mathrm{M_{bulk}}$, and U_{p} is the peak potential for adsorption on the supporting substrate.

We have tried to correlate the ΔU_{p} value with physical parameters pertinent to the system that might semiquantitatively explain the energy gain for the energetically favored deposition (UPD) compared to bulk deposition. Because μ (in atomic units) is defined as the Gibbs free energy that can be absorbed/released owing to the change of a metal atom, one might assume that the binding energy (ΔG_{BE}), which also reflects the energy required to dissociate a metal atom from the substrate atom, should have a positive correlation with μ:

$$\Delta\mu_{\mathrm{S/M}} = k\Delta G_{\mathrm{BE}} + \mu_0 = z\mathrm{e}_0\Delta U_{\mathrm{p}} \qquad (12)$$

Thus,

$$\Delta U_{\mathrm{p}} = \frac{k}{z\mathrm{e}_0}\Delta G_{\mathrm{BE}} + \frac{\mu_0}{z\mathrm{e}_0} \qquad (13)$$

where the slope $\frac{k}{z\mathrm{e}_0}$ and intercept $\frac{\mu_0}{z\mathrm{e}_0}$ are only related to the intrinsic nature of the deposited metal. Combining available experimental (Supplementary Fig. 13) and DFT calculation data (Supplementary Table 6), we note that ΔU_{p} is linearly proportional to the absolute value of single-atom–support binding energy (ΔG_{BE}; Supplementary Fig. 15a and Supplementary Table 7).

The linear relationship connects the difference in the binding energy (metal–support) between a metal adatom bound to a substrate atom and one bound in bulk material with the difference in electron energies at the respective electrode potentials, in order to establish equilibrium conditions for the reactions (Supplementary Fig. 15b). The site-specific UPD of single-atom metals can be influenced by factors such as potential window, nature of substrate and adsorption of ions. The process can be reasoned as follows: (i) solvated metal ions move from the diffuse layer to the reaction zone, getting rid of the solvation sheath, and (ii) electron transfer from the substrate to the metal ions, leading to the subsequent formation of metal-substrate bond (Supplementary Fig. 15c). During this process, the formation of metal-substrate bond involves knocking off the adsorbed solvent dipoles from the deposition sites of the

substrate. Note that the substrate is solvated, we should also consider the nature of the arrangement of solvent dipoles at the substrate surface (Supplementary Fig. 15c).

Supplementary Note 5. Plausible mechanism for the interaction between copper ions and ce-MoS_2

When discussing the growth mechanism of UPD of Cu on ce-MoS_2, we first exclude the possibility of functionalization by physisorption (Supplementary Fig. 18). Instead, we postulate that surface S atoms of ce-MoS_2 first coordinate the Lewis acidic metal prior to UPD (Supplementary Fig. 19a). Each MoS_2 layer consists of S—Mo—S structures with van der Waals forces existing between the two neighboring S layers. MoS_2 can provide Lewis base character because interfacial activated S atoms have lone pair electrons for potential functionalization by Lewis acid-base chemistry. Here, we studied the interaction between Cu^{2+} (a typical Lewis acid) and MoS_2 (a Lewis base). According to molecular orbital theory, each S atom in 1T-MoS_2 has a tetrahedral orbital configuration attributed to sp^3 hybridization ([Ne]$3s^2 3p^4$). With respect to the four sp^3 orbitals, three constitute Mo—S bonds, whereas the forth orbital is fully occupied by lone pair electrons (Supplementary Fig. 19a). Lewis acid Cu^{2+} ions, possessing empty electron orbitals, can accept the lone pair electrons and form a quasi-stable coordinate intermediate. The lone pair electrons of S atoms couple with the empty orbitals of Cu^{2+} and form the coordination intermediate complex $[Cu^{2+}{}_n(MoS_2)](SO_4{}^{2-})_n$.

As shown by XPS analysis (Supplementary Fig. 19d), a characteristic peak for Cu 2p appears, which can be unambiguously ascribed to the adsorption of Cu^{2+} ions on the ce-MoS_2 nanosheets[9]. The binding energies (953.5 and 933.8 eV) for the adsorbed Cu ions are located in the range between Cu^0 and Cu^{II},[10,11] suggesting partial electron donation from the lone pair electrons of the S atom. As a result of electronic interaction with the Lewis acidic metal atoms, the surface S atom donors become more electropositive, resulting in a slightly broader band and higher photoelectron emission energy (Supplementary Fig. 19c), which in turn confirms this effect. In the UV-visible spectrum, the peak in the near-UV region corresponding to ce-MoS_2 is also redshifted (Supplementary Fig. 19e). These changes are consistent with the dominant mechanism as proposed above, in which the formal positive charge on the S atoms facilitate the electron extraction.

Then, an applied potential served as an electron donor to reduce *in situ* the Cu^{2+} ions to Cu^0 adatoms on MoS_2. Owing to the driving force of UPD that is determined by the specific affinity between metal adatoms and the substrate atoms, the reaction was terminated, and no additional Cu^{2+} ions were able to bind to MoS_2 because the interfacial S atoms had fully reacted, leading to the construction of a stable single-atom model (identified by DFT calculations, Supplementary Table 6, Supplementary Fig. 19b).

Supplementary Note 6. HER mechanism

It is well recognized that there are two possible mechanisms for the HER in acidic solution (Supplementary Fig. 22)[12], both starting with reductive proton adsorption (S-14). The Volmer–Heyrovsky mechanism (S-14 and S-15) follows that with reduction of a second proton at the same site and release of a hydrogen molecule (two-electron process). In the Volmer–Tafel mechanism (S-14 and S-16), proton adsorption is followed immediately by the surface combination of two adsorbed hydrogen atoms and the release of a hydrogen molecule (one-electron process).

$H_3O^+ + e^- \rightarrow H_{ads} + H_2O$ (14)

$H_{ads} + H_3O^+ + e^- \rightarrow H_2 + H_2O$ (15)

$H_{ads} + H_{ads} \rightarrow H_2$ (16)

Supplementary Note 7. Calculation of TOF values

Pt-SAs/MoS_2:

$$n = \frac{6.25 \times 10^{-4} \times 5.1\% \times 10^{-3}}{195} = 1.64 \times 10^{-10}\ \mathrm{mol}$$

$$\mathrm{TOF(@0.05\ V)} = \frac{I}{2F \times n} = \frac{5.163 \times 0.07065 \times 10^{-3}}{2 \times 96500 \times 1.64 \times 10^{-10}} = 11.52\ \mathrm{s^{-1}}$$

$$\mathrm{TOF(@0.10\ V)} = \frac{I}{2F \times n} = \frac{21.21 \times 0.07065 \times 10^{-3}}{2 \times 96500 \times 1.64 \times 10^{-10}} = 47.3\ \mathrm{s^{-1}}$$

$$\mathrm{TOF(@0.15\ V)} = \frac{I}{2F \times n} = \frac{46.33 \times 0.07065 \times 10^{-3}}{2 \times 96500 \times 1.64 \times 10^{-10}} = 103.37\ \mathrm{s^{-1}}$$

$$\mathrm{TOF(@0.20\ V)} = \frac{I}{2F \times n} = \frac{78.55 \times 0.07065 \times 10^{-3}}{2 \times 96500 \times 1.64 \times 10^{-10}} = 175.27\ \mathrm{s^{-1}}$$

Pt-SAs/WS_2:

$$n = \frac{7.15 \times 10^{-4} \times 4.1\% \times 10^{-3}}{195} = 1.50 \times 10^{-10}\ \mathrm{mol}$$

$$\mathrm{TOF(@0.20\ V)} = \frac{I}{2F \times n} = \frac{112 \times 0.07065 \times 10^{-3}}{2 \times 96500 \times 1.50 \times 10^{-10}} = 272.9\ \mathrm{s^{-1}}$$

Pt-SAs/$MoSe_2$:

$$n = \frac{5.5 \times 10^{-4} \times 4.7\% \times 10^{-3}}{195} = 1.33 \times 10^{-10}\ \mathrm{mol}$$

$$\mathrm{TOF(@0.20\ V)} = \frac{I}{2F \times n} = \frac{59.23 \times 0.07065 \times 10^{-3}}{2 \times 96500 \times 1.33 \times 10^{-10}} = 163.1\ \mathrm{s^{-1}}$$

Pd-SAs/MoS_2:

$$n = \frac{6.25 \times 10^{-4} \times 2.83\% \times 10^{-3}}{106.42} = 1.66 \times 10^{-10}\ \mathrm{mol}$$

$$\mathrm{TOF(@0.20\ V)} = \frac{I}{2F \times n} = \frac{45.78 \times 0.07065 \times 10^{-3}}{2 \times 96500 \times 1.66 \times 10^{-10}} = 101.0\ \mathrm{s^{-1}}$$

Supplementary Note 8. Proposed HER mechanism on Pt-SAs/MoS_2

The Tafel slope is an inherent property of electrocatalytic materials and is a useful indicator of the rate-limiting step for reactions involving electron transfer. The Pt-SAs/MoS_2 catalyst yields a Tafel slope of 31 mV dec^{-1}, which is close to the value of commercial Pt/C (32 mV dec^{-1}, theoretical value: 30 mV dec^{-1}). The seemingly identical Tafel behavior of commercial Pt/C and Pt-SAs/MoS_2 is not necessarily an indicator that the exact same HER pathway is followed. Our experimental and theoretical data (XANES, XPS, and Bader charges analysis) have shown that single Pt atoms are positively charged and that the S atom obtains the electron, leading to a much higher total unoccupied density of Pt 5*d* states. During H chemisorption, the 5*d* orbitals of the Pt atoms interact strongly with the 1s orbital of the H atoms, leading to electron pairing and hydride formation[13,14]. In this case, we reason that atomic-scale tailoring should unconventionally modulate the adsorption state of hydrogen atoms on the Pt single atom of Pt-SAs/MoS_2.

For Pt-based catalysts with high hydrogen coverage, two H^+ ions in the solution are usually reduced into a H_2 gas molecule through two initial Volmer steps ($H^+ + e^- \rightarrow H_{ads}$) and a subsequent Tafel step ($2H_{ads} \rightarrow H_2$). During the initial Volmer steps, adsorbed H^+ ions are chemically bonded to the Pt surface in the form of Pt–H bonds. During the Tafel reaction, for traditional Pt nanocrystals, the two protons bind to two adjacent Pt atoms, combine, and generate a H_2 molecule (Supplementary Fig. 29a), whereas for Pt SA, the two protons bind to a single Pt atom, combine and produce H_2 (Supplementary Fig. 29b and c).

To demonstrate the underlying type of mechanism dominant in the Pt-SAs/MoS_2 system, we further performed a computational study on single-Pt-atom catalysts to gain more detailed insights into the HER process (Supplementary Fig. 29d). After taking the number of unoccupied Pt 5*d* orbitals and the steric hindrance of interacting H atoms into consideration, we conclude that the maximum number of adsorbed H atoms in this Pt-SAs/MoS_2 system is six per Pt atom. The strengths of hydrogen adsorption exhibit an overall decreased trend with an increase in the number of adsorbed H atoms, resulting in a minimum value if six H atoms are adsorbed (Supplementary Fig. 29d). The higher H coverage on a Pt atom favors the Tafel reaction because the increase in H coverage efficiently decreases the adsorption strength of H_2 molecules[15]. As the number of interacting H atoms increases, the formation of Pt—H bonds is also influenced by the electrostatic repulsion between the neighboring H atoms. The interaction between the Pt and H atoms largely depends on the number of interacting H atoms, and the

complex distinct orbitals for bond formation become a compromise between the H–H electrostatic repulsion and the orbital interaction between Pt and H. It should be noted that if three H atoms are adsorbed onto a single Pt atom, a stable triangular structure of H atoms with the highest binding energy is achieved, from which no H_2 dimer is formed. Interestingly, each triangular vertex H atom would serve as a catalytic active site for the subsequent hydrogen adsorption to easily form H_2 dimers. For example, as the number of H atoms reaches five, two of the H atoms form an H_2 dimer and are automatically released from a single Pt atom, leaving the triangular structure of H atoms remaining on that Pt atom. Finally, as the number of H atoms increases to six, three H_2 dimers are automatically released from the single Pt atom. The increased number of catalytic active sites of single Pt atoms for H adsorption, as well as the decreased adsorption energy of the product H_2, decreases the effective barrier in the overall kinetics, which determines the fast HER kinetics we experimentally observed.

Although a systematic experimental kinetic investigation is beyond the scope of the current work, the likely HER mechanism of hydrogen recombination and desorption for single-atom Pt on the ce-MoS_2 is proposed using DFT calculations, with an attempt to obtain insights into the kinetics of HER.

Supplementary Note 9. Comparison between the previously reported electrodeposition method and SSED for ADMC synthesis

Electrodeposition offers a facile, controllable and room-temperature method for reducing metal ions into their elemental states. In this case, an efficient electrodeposition method has been widely used for single-atom synthesis in recent years[16-20]. The cathodic deposition of single-atom metals on a working electrode can be achieved by anodic dissolution of a bulk metal foil electrode as a counter electrode under acidic conditions using a three-electrode configuration (Supplementary Fig. 30a). However, the potential cycling process is usually completed within hours (at least 10 h) and is less controllable, which results in the formation of nanoclusters or nanoparticles at longer cycling times.

Recently, Zeng's group have reported a universal and rapid electrodeposition approach for the fabrication of single-atom metals[21]. The depositions can be both cathodically and anodically conducted (C, A-ED) for synthesis of single-atom metals with distinct electronic states, which holds great promises for various catalytic reactions. They proposed that the electrodeposition process resembles the molecular nucleation mechanism. The upper limit of mass loading for single-atom metals cannot exceed the minimum supersaturation level, otherwise single-atom metal tends to nucleate (Supplementary Fig. 30b). Thus, single-atom synthesis can be realized by controlling the metal precursor concentration and deposition time.

In our work, we developed an "intelligent" methodology for the single-atom growth by site-specific electrodeposition which is capable of automatically terminating the aggregation of metal atoms (Supplementary Fig. 30c). Such intrinsically self-terminating effect distinguishes our site-specific UPD method from other previously reported electrodeposition method for single-atom synthesis. We show that the site-specific UPD method can be used to produce high-loading single-atom metals without the consideration of the high metal precursors or long deposition time (Supplementary Table 10), which might seriously cause atom aggregation in other electrodeposition methods. After the formation of thermodynamically favorable metal–support bonds, the sequential formation of metal–metal bond is forbidden at the UPD potential, restricting it to the single-atom metal. In our design, two requisite factors should be considered: (1) identifying electrically conductive substrate materials, which consist of isolated active sites that possess lone pair electrons and suitable electronegativity for the UPD of single atoms; (2) choosing suitable applied electrodeposition potential restricted to UPD region, at which metal–support bonding predominates over metallic bonding. Our site-specific UPD method for single-

atom synthesis can be rapidly completed on a timescale of seconds to minutes. Additionally, we confirm that single-atom metals are straddled atop Mo by coordinating with three nearest neighboring S of Mo (see HADDF-STEM image, EXAFS data, and DFT simulations), which shows distinct from depositing site of vacancies/edges/defects into the lattice reported by previous works[16-21].

Supplementary Figures

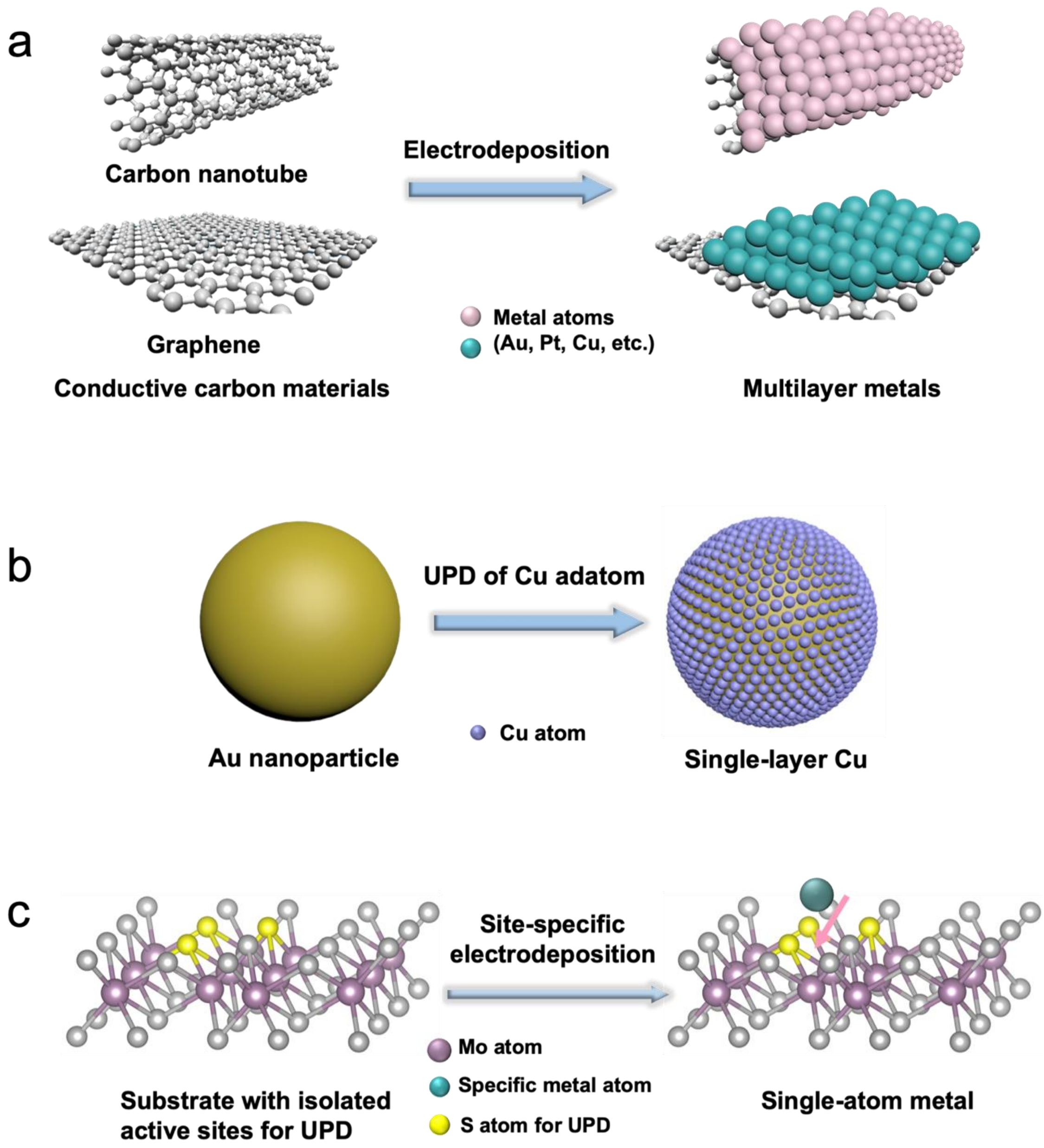

Supplementary Figure 1. Examples of metal preparation by electrodeposition. Schematic representations of examples of the electrodeposition of multilayer metals (a), single-layer metals (b), and single-atom metals (c). A detailed description is given in Supplementary Note 1.

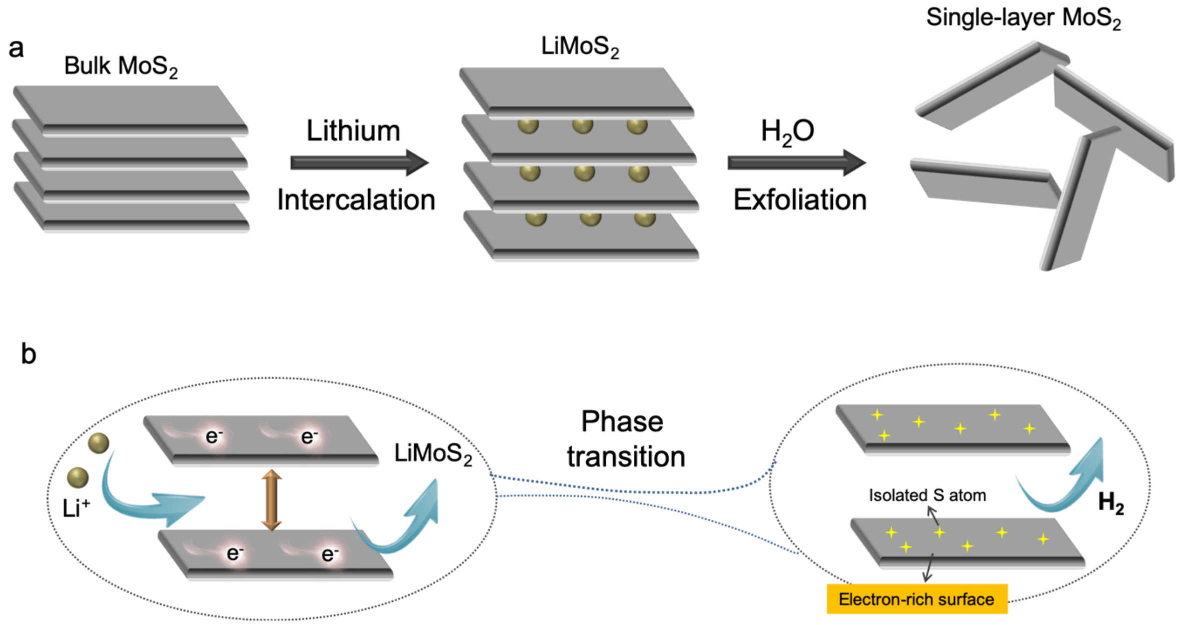

Supplementary Figure 2. (a) Chemical exfoliation of bulk MoS_2 through lithium intercalation. (b) Phase transition and creation of isolated S atoms during chemical exfoliation. Lithium intercalation is often used to exfoliate layered crystals[22-24]. During lithium intercalation, bulk MoS_2 is first intercalated with lithium to form Li_xMoS_2 by reacting MoS_2 powder with *n*-butyllithium [$x(n\text{-}C_4H_9Li) + MoS_2 \rightarrow Li_xMoS_2 + x/2\ C_8H_{18}$, $x \geq 1$]. During this process, the insertion of Li^+ ions is tantamount to the injection of massive quantities of electrons into the MoS_2 crystal, resulting in a phase transition from the semiconducting 2H phase to the metallic 1T phase. Then, the intercalated Li_xMoS_2 can be easily exfoliated in water through forced hydration to release H_2, which enlarges the layer distance between MoS_2 nanosheets ($Li_xMoS_2 + H_2O \rightarrow [MoS_2]^{x-} + xLi^+$). The chemical exfoliation method results in a water-dispersible MoS_2 suspension (ce-MoS_2), which maintains good colloidal stability (Supplementary Fig. 3). Note that chemically exfoliation endows MoS_2 with more S vacancies[25], which also efficiently separate S atoms for single-atom growth. It has been previously reported that S atoms of the electron-rich MoS_2 nanosheets have high reactivity towards surface covalent functionalization and some catalytic reactions. For instance, basal-plane functionalization at the S atoms of ce-MoS_2 has been realized by reacting ce-MoS_2 with strong electrophiles (e.g., diazonium salts and organohalides)[22,26,27]. Furthermore, recent experimental and computational results have demonstrated that the catalytic active sites of ce-MoS_2 (1T-phase) nanosheets for the hydrogen evolution reaction (HER) are mainly located on the basal plane, and the electron-rich S atoms might be the adsorption sites for hydrogen atoms[28,29].

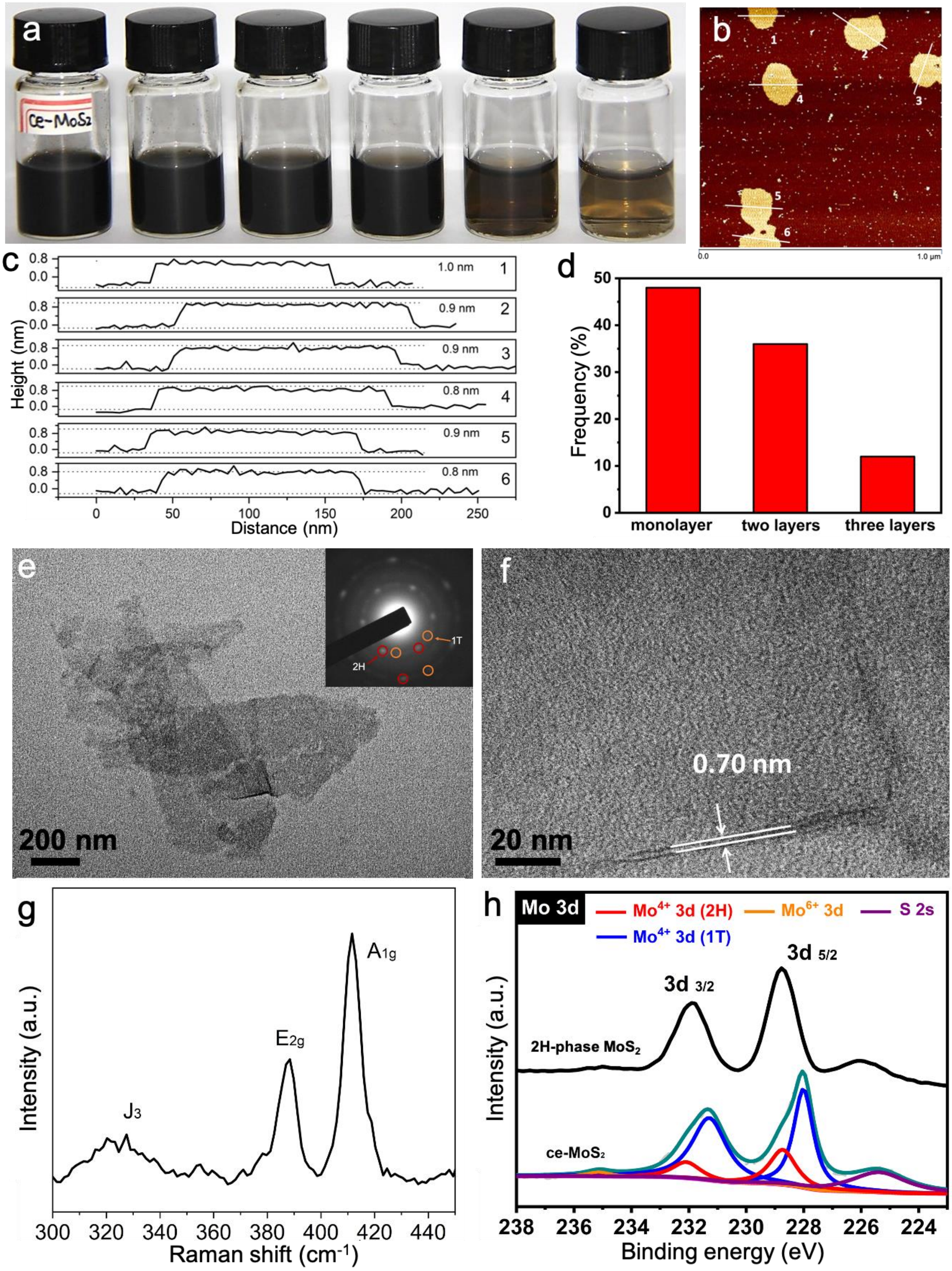

Supplementary Figure 3. Physical characterization of ce-MoS_2 nanosheets. (a) Photographs of readily dispersed ce-MoS_2 nanosheets suspended in water at different concentrations (left to right: 1, 0.5, 0.25, 0.125, 0.0625, and 0.0313 mg mL^{-1}). The water-dispersible ce-MoS_2 suspension appears yellow-green after dilution with excellent colloidal

stability. (b) AFM image of uniformly spin-coated ce-MoS_2 nanosheets. (c) Height profiles of the regions of interest indicated in the AFM image. (d) Histogram of the layer distribution of ce-MoS_2 nanosheets. A statistical analysis of 30 flakes obtained by the lithium intercalation method indicated that the ce-MoS_2 nanosheets contain flakes in which 48% are single layers, 35% have two layers, 13% have three layers, and the remainder have a much lower concentration of stacked layers. Note that the single-layer thickness of ce-MoS_2 is slightly larger than the value of 0.65–0.70 (nm) reported for MoS_2 single layers obtained by mechanical exfoliation[30,31]. This discrepancy can be attributed to the surface corrugation of ce-MoS_2 caused by distortions or the presence of adsorbed molecules under aerobic conditions[124,32]. (e) The TEM image shows that the majority of ultrathin ce-MoS_2 nanosheets have lateral dimensions of 100–1000 nm. Inset: the corresponding selected area electron diffraction (SAED) pattern of ce-MoS_2 nanosheets shows a hexagonal spot pattern ascribed to 2H phase (red circle) and another hexagonal spot at 30° between the hexagonal spots (orange circle) assigned to the 1T phase, which is indicative of a mixed phase (1T and 2H) and consistent with previously reported ce-MoS_2[23]. (f) A zoom of panel a shows an interplanar spacing of 0.70 nm, which is assigned to the *d*-spacing of the (002) plane of hexagonal MoS_2. (g) Raman spectrum of the ce-MoS_2 sample. The new emergence of peak at ca. 328 cm^{-1}, which is assigned to the J_3 mode, confirms the formation of 1T phase[33]. (h) Mo 3d XPS spectra of the ce-MoS_2 and 2H-phase MoS_2. The Mo 3d XPS peaks of 1T-phase ce-MoS_2 are deconvoluted into three components: 1) the doublet peaks at 232.1 and 228.7 eV correspond to Mo^{4+} $3d_{3/2}$ and Mo^{4+} $3d_{5/2}$ of the 2H-phase MoS_2, respectively; 2) the doublet peaks at 231.2 and 228.0 eV are ascribed to Mo^{4+} $3d_{3/2}$ and Mo^{4+} $3d_{5/2}$ of the 1T-phase MoS_2, respectively; and 3) the weakly intense peak at 235.1 eV corresponds to a higher oxidation state, Mo^{6+}. The deconvolutions of Mo 3d peak fitting demonstrates the predominance of the metallic 1T phase in the ce-MoS_2 sample (~75%)[34].

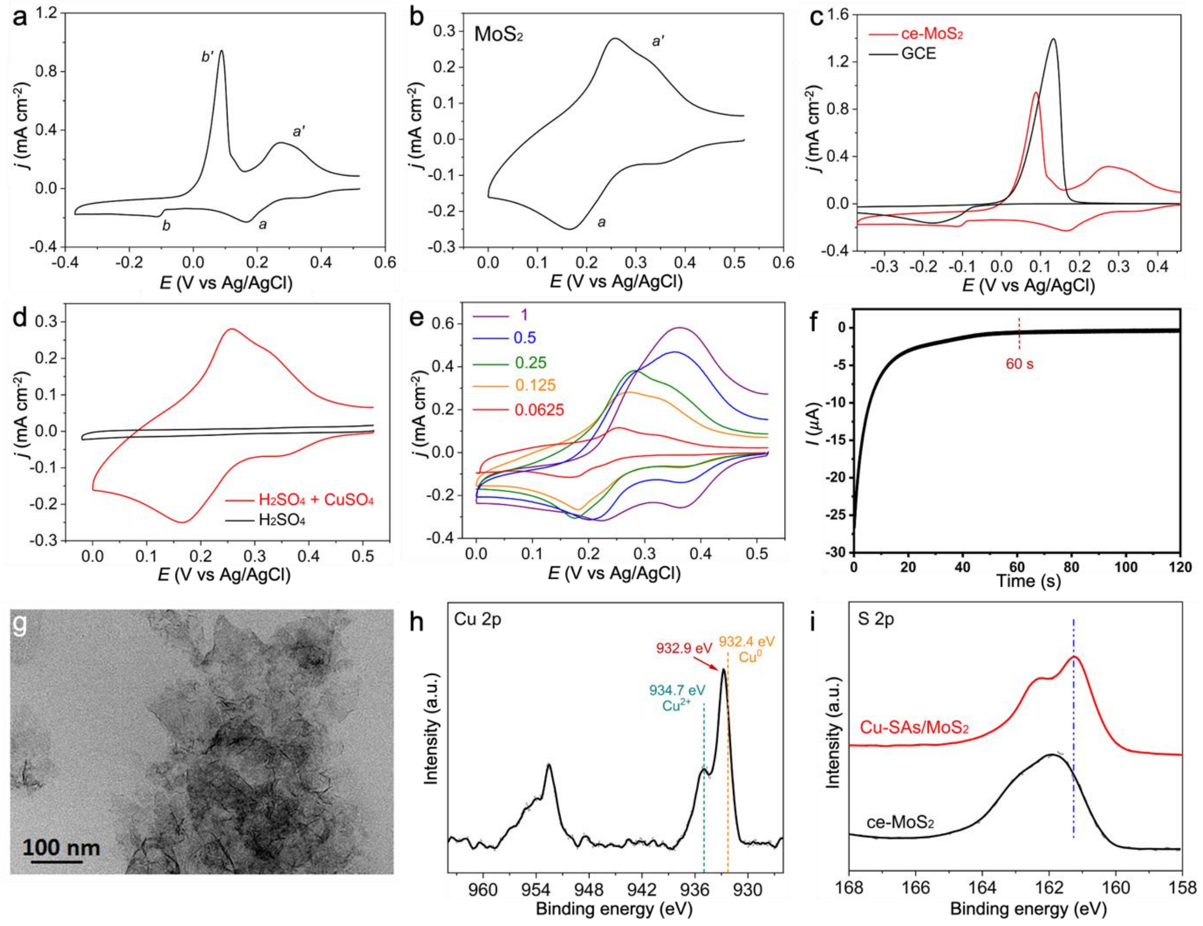

Supplementary Figure 4. Electrochemical and physical characterization of the process of Cu UPD on the ce-MoS_2 nanosheets. (a) Cyclic voltammogram (CV) of a ce-MoS_2-modified GCE in an Ar-saturated 0.1 M H_2SO_4 solution containing 2 mM $CuSO_4$ at a scan rate of 20 mV s^{-1}. The CV displays two notable cathodic peaks (*a* and *b*) at approximately +0.15 and −0.10 V, respectively. Peak *a* is attributed to Cu UPD, whereas peak *b* corresponds to the bulk deposition of Cu. The two anodic peaks (*b′* and *a′*) that appeared in the positive potential scan arise from dissolution of the bulk Cu deposit and of Cu deposited by UPD, respectively. (b) CV restricted to the Cu UPD region. (c) CVs of ce-MoS_2 and GCE in an Ar-saturated 0.1 M H_2SO_4 solution containing 2 mM $CuSO_4$ at a scan rate of 20 mV s^{-1}. (d) CVs of ce-MoS_2 in an Ar-saturated 0.1 M H_2SO_4 solution containing 2 mM $CuSO_4$ (red line) or 0.1 M H_2SO_4 solution (black line) at a scan rate of 20 mV s^{-1}. (e) Optimization for the loading concentration (indicated in mg mL^{-1}) of ce-MoS_2 on a GCE. The optimization results are shown in detail in Supplementary Table 1. (f) Chronoamperometry curve of Cu UPD on ce-MoS_2 nanosheets. An experiment with a running time of 120 s was conducted with a constant applied potential of 0.1 V (vs Ag/AgCl) in 0.1 M H_2SO_4 solution containing 2 mM $CuSO_4$. On the basis of the deposition

current response, the UPD process mainly occurs within approximately 60 s because the current gradually approaches zero. Therefore, a 120 s running time for UPD was deemed sufficient. (g) Conventional TEM image of a Cu-SAs/MoS_2 sample. (h) Cu 2p and (i) S 2p XPS spectra of a Cu-SAs/MoS_2 sample. The main Cu $2p_{3/2}$ peak of Cu-SAs/MoS_2 at 932.9 eV is located between Cu^0 (932.4 eV) and Cu^{2+} (934.7 eV)[10,11], indicative of Cu species with partially positive charge owing to the electronic interaction between single Cu atom and ce-MoS_2. The absence of Cu 2p satellite peaks (typically at ca. 940–944 eV) further confirms the ionic nature of Cu species in Cu-SAs/MoS_2[35]. Simultaneously, the average binding energy of S 2p decreases and its shape becomes slightly more pronounced after functionalization of Cu atoms compared to that of pure ce-MoS_2, which confirms the attachment of Cu atoms onto the S atoms.

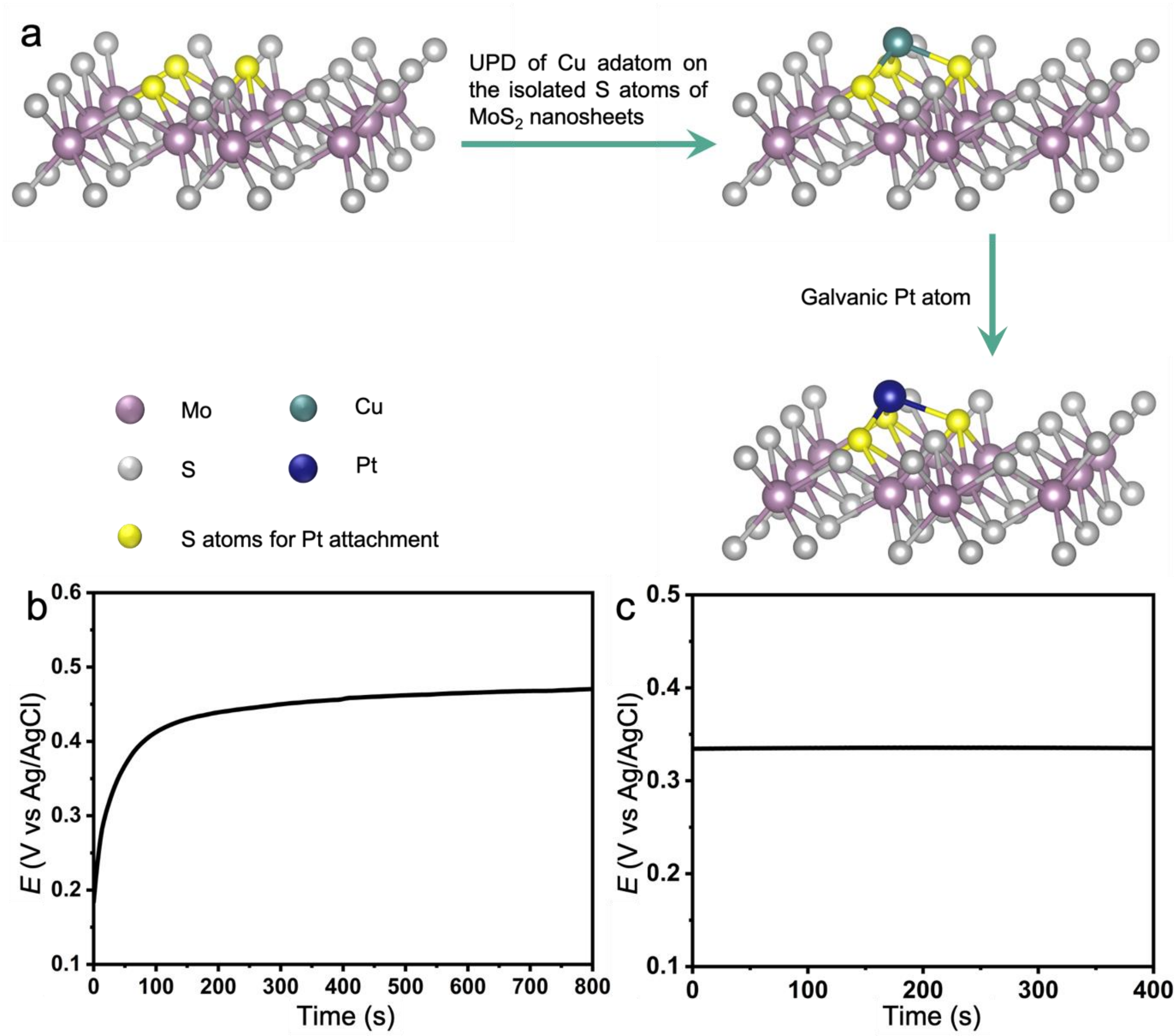

Supplementary Figure 5. The preparation of Pt-SAs/MoS_2. (a) Schematic illustration of synthesis process. After site-specific UPD of Cu adatoms on the S atoms, galvanic replacement of Cu adatoms with $PtCl_4^{2-}$ at the open-circuit potential is conducted to produce Pt-SAs/MoS_2. (b) Electroless galvanic replacement of Cu adatoms with Pt^{II} at the open-circuit potential. Based on the potential response, the potential gradually changes over 800 s from 0.17 to 0.47 V. This result indicates that the 20 min reaction time is sufficient for the complete galvanic replacement of Cu with Pt. (c) Open-circuit potential of ce-MoS_2-modified GCE immersed in K_2PtCl_4 solution. No obvious changes in current were observed. The result excludes the possibility of the direct reduction of $PtCl_4^{2-}$ to single Pt atoms by ce-MoS_2, indicating that the Cu UPD process is indispensable for successful fabrication of Pt SA.

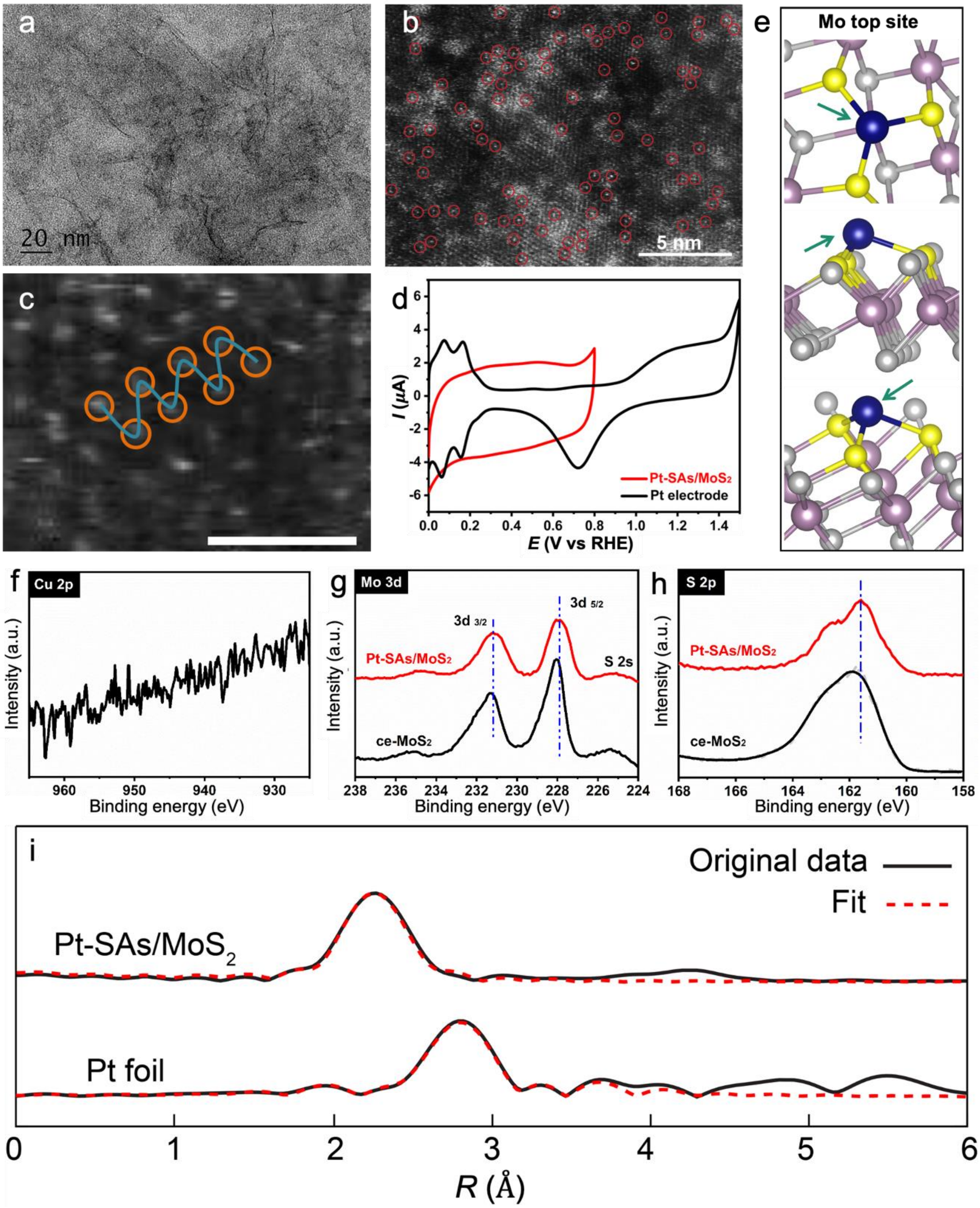

Supplementary Figure 6. Characterizing the morphology and structure of Pt-SAs/MoS_2. (a) Conventional TEM images of a freshly prepared Pt-SAs/MoS_2 sample (scale bar: 20 nm). No obvious clusters or nanoparticles were found in Pt-SAs/MoS_2, implying that most of the Pt exists in the atomically dispersed form. (b) HAADF-STEM image of Pt-SAs/MoS_2. (c) Magnified HAADF-STEM image of freshly prepared Pt-SAs/MoS_2. The zigzag pattern in Pt-SAs/MoS_2 was directly observed, which indicated the slightly distorted 1T structure after

functionalization of Pt atoms. (d) CVs for Pt-SAs/MoS_2 and Pt electrodes in Ar-saturated 0.5 M H_2SO_4 solution with a scan rate of 50 mV s^{-1}. The scanning potential for the cyclic voltammetry of Pt-SAs/MoS_2 could not exceed 0.75 V (vs RHE) because MoS_2 tends to oxidize to MoO_3 at higher potentials[36]. Compared with the standard freshly polished Pt electrode, Pt-SAs/MoS_2 shows no obvious characteristic redox peaks in the regions of Pt–H adsorption/desorption, indicative of the ultralow Pt loading beyond the detection limit of cyclic voltammetry. (e) Top, side and perspective views (up to bottom, respectively) of DFT-calculated geometries of Pt-SAs/MoS_2 with the Pt atom on the Mo top site. The color code is the same as in Supplementary Fig. 5. (f) Cu 2p XPS spectrum of Pt-SAs/MoS_2 sample. Mo 3d (g) and S 2p (h) XPS spectra of Pt-SAs/MoS_2 and ce-MoS_2. The surface of Pt-SAs/MoS_2 was observed to be free of Cu, indicating the galvanic replacement reaction was completed. With the decoration of Pt single atoms, the average binding energies of Mo 3d and S 2p decrease, whereas the binding energy of Pt 4f significantly increases (Fig. 2g). Compared to that of Pt nanoparticles, the core-level binding energy of Pt 4f for Pt-SAs/MoS_2 is higher, owing to the formation of Pt—S bonds upon functionalization. Electron transfer from Pt to MoS_2 reduces the electron density in the Pt outer shells (5d and 6s orbitals), and then, a stronger attractive interaction is applied to the inner shell (4f), resulting in XPS peaks at higher binding energies. Simultaneously, the S 2p peak of Pt-SAs/MoS_2 becomes slightly more intense relative to that in the spectrum of ce-MoS_2, suggesting the attachment of single Pt atoms to the S atoms. The absence of peaks corresponding to Pt—Pt bonds at 71.0 and 74.4 eV further confirms that no Pt nanoparticles or crystalline phases were formed. These results demonstrate the mutual interactions between the isolated Pt atoms and the ce-MoS_2 support, along with the transfer of electrons from Pt to ce-MoS_2. (i) First-shell fitting for EXAFS profiles of the Fourier transform at Pt L_3-edge (fitting parameters shown in Supplementary Table 4).

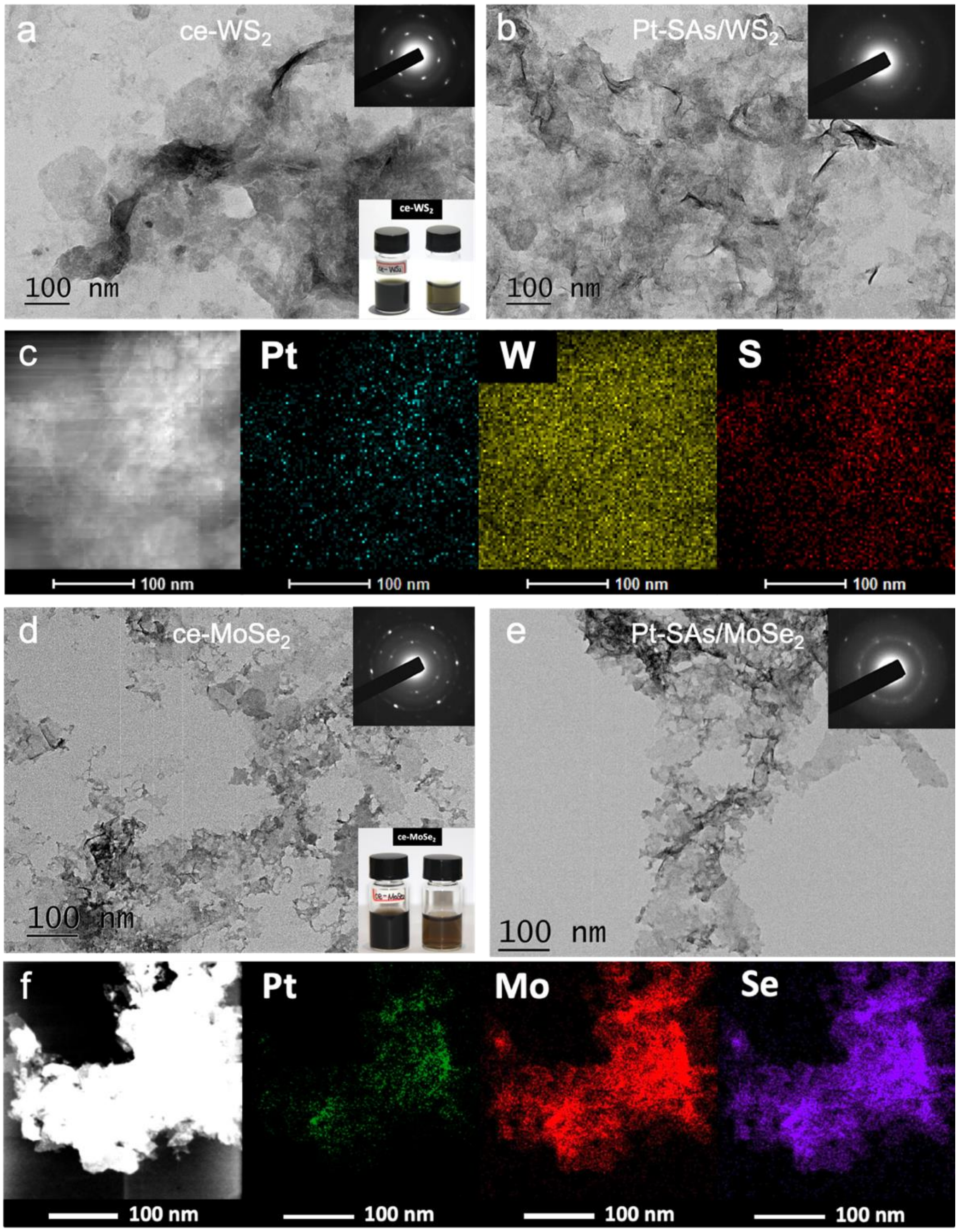

Supplementary Figure 7. Characterizing the morphology of ce-WS_2 and ce-$MoSe_2$ nanosheets before and after functionalization with single Pt atoms. (a) TEM image of freshly prepared ce-WS_2 nanosheets; scale bar: 100 nm. The upper-right inset shows the corresponding SAED pattern. The lower-right inset shows the photograph of well-dispersed ce-WS_2 nanosheet suspensions in water at concentrations of 0.87 mg mL^{-1} (left) and 0.11 mg mL^{-1} (right). (b) Conventional TEM image of the freshly prepared Pt-SAs/WS_2; scale bar: 100 nm. Inset: the corresponding SAED pattern. No obvious clusters or nanoparticles were found

in Pt-SAs/WS_2, implying that most of the Pt exists in the atomically dispersed form. (c) EDX mapping images of Pt, W, and S elements. (d) TEM image of freshly prepared ce-$MoSe_2$ nanosheets; scale bar: 100 nm. The upper-right inset shows the corresponding SAED pattern. The lower-right inset shows the photograph of well-dispersed ce-$MoSe_2$ nanosheet suspensions in water at concentrations of 0.148 mg mL^{-1} (left) and 0.037 mg mL^{-1} (right). (e) Conventional TEM image of the freshly prepared Pt-SAs/$MoSe_2$; scale bar: 100 nm. Inset: the corresponding SAED pattern. No obvious clusters or nanoparticles were found in Pt-SAs/$MoSe_2$, implying that most of the Pt exists in the atomically dispersed form. (f) EDX mapping images of Pt, Mo, and Se elements.

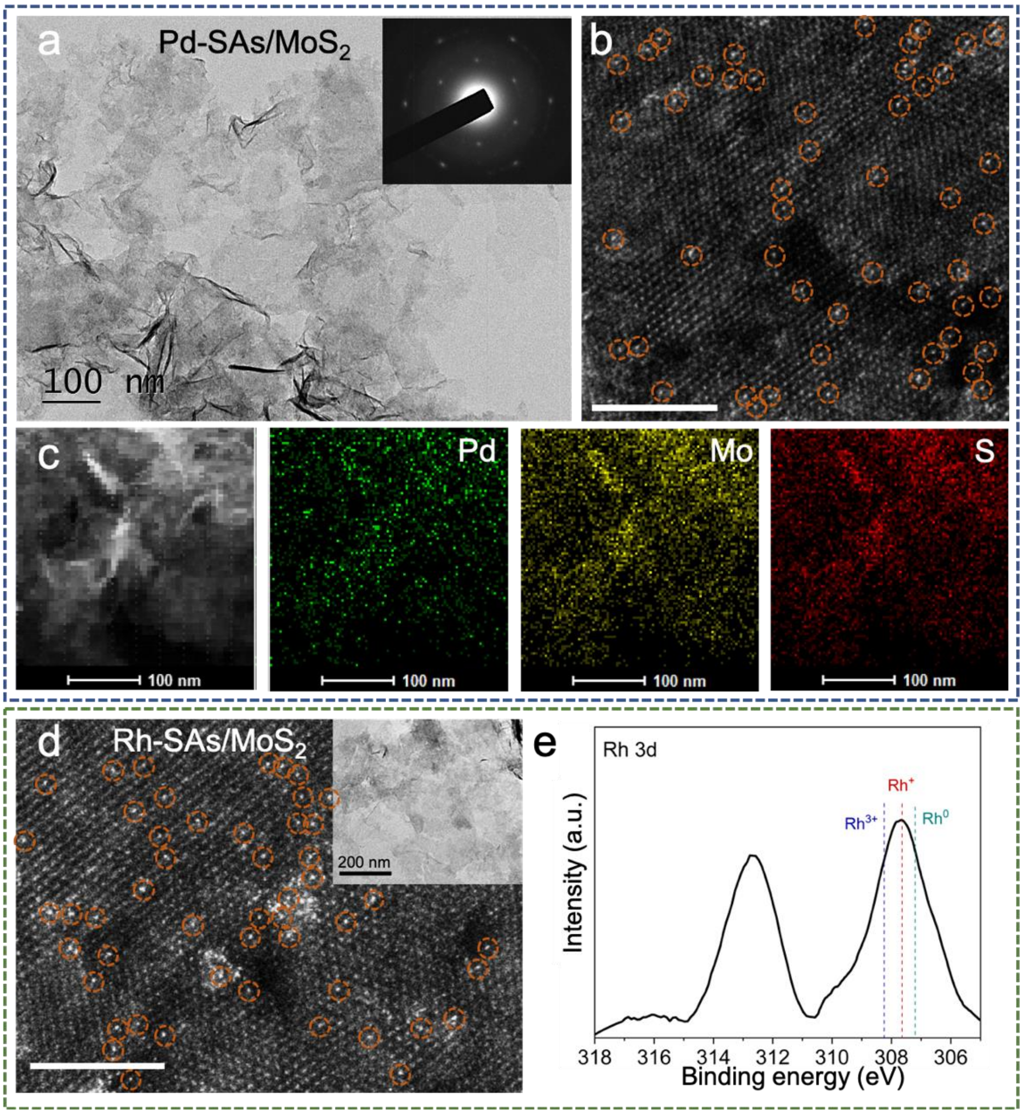

Supplementary Figure 8. Characterizing the morphology of Pd-SAs/MoS_2 and Rh-SAs/MoS_2. (a) A conventional TEM image of a freshly prepared Pd-SAs/MoS_2 sample. Inset: the corresponding SAED pattern. No obvious clusters or nanoparticles are found in Pd-SAs/MoS_2, implying that most of the Pd exists in the atomically dispersed form. (b) HAADF-STEM image of Pd-SAs/MoS_2 (scale bar: 5 nm). (c) EDX mapping images of Pd, Mo, and S elements. (d) HAADF-STEM image of Rh-SAs/MoS_2 (scale bar: 5 nm). Inset shows the conventional TEM images of a freshly prepared Rh-SAs/MoS_2 sample. (e) Rh 3d XPS spectrum of Rh-SAs/MoS_2. The core-level binding energy of Rh 3d 5/2 in Rh-SAs/MoS_2 is located at ca. 307.6 eV. Considering that the XPS Rh^0 peak is located at ca. 307.2 eV, the Rh^+ peak at ca. 307.5 eV, and the Rh^{3+} peak at ca. 308.3 eV[37,38], the Rh atoms in Rh-SAs/MoS_2 are positively charged and have an oxidation state of ca. +1.

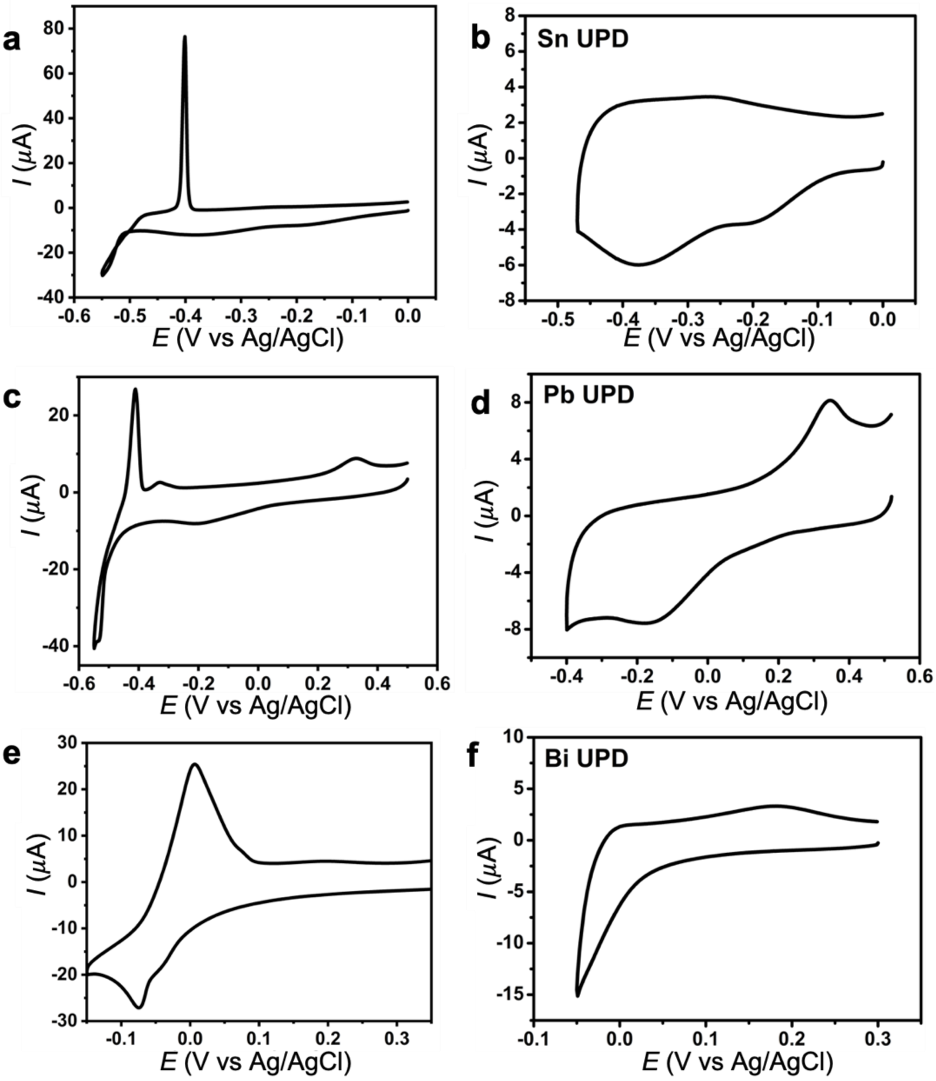

Supplementary Figure 9. Various single-atom metals underpotentially deposited on ce-MoS_2. (a, c, e) CVs of a ce-MoS_2-modified GCE for the UPD of different metal atoms in Ar-saturated solutions (1 mM $Pb(NO_3)_2$ + 0.1 M $HClO_4$; 1 mM Sn^{2+} + 0.2 M $HClO_4$; 1 mM $Bi(NO_3)_3$ + 0.5 M H_2SO_4) at a scan rate of 20 mV s^{-1}. (b, d, f) CVs of the UPD region for the different metals. Pb, Sn, and Bi atoms can be readily underpotentially deposited on the ce-MoS_2 nanosheets. According to reactivity series of metals (Co>Ni>Sn>Pb>H_2>Bi>Cu>Hg), the metals (Sn, Pb, Bi, and Cu) underpotentially deposited on ce-MoS_2 incidentally possess the similar reactivity as H_2. Recent experimental and computational results have demonstrated that the catalytic sites of ce-MoS_2 nanosheets for HER are mainly the electron-rich S atoms[28,29], which could be the adsorption site for H atoms. In that case, we hypothesize that the electron-rich S atoms have adsorption energies that favor the binding of these metal atoms possessing the similar reactivity as H_2.

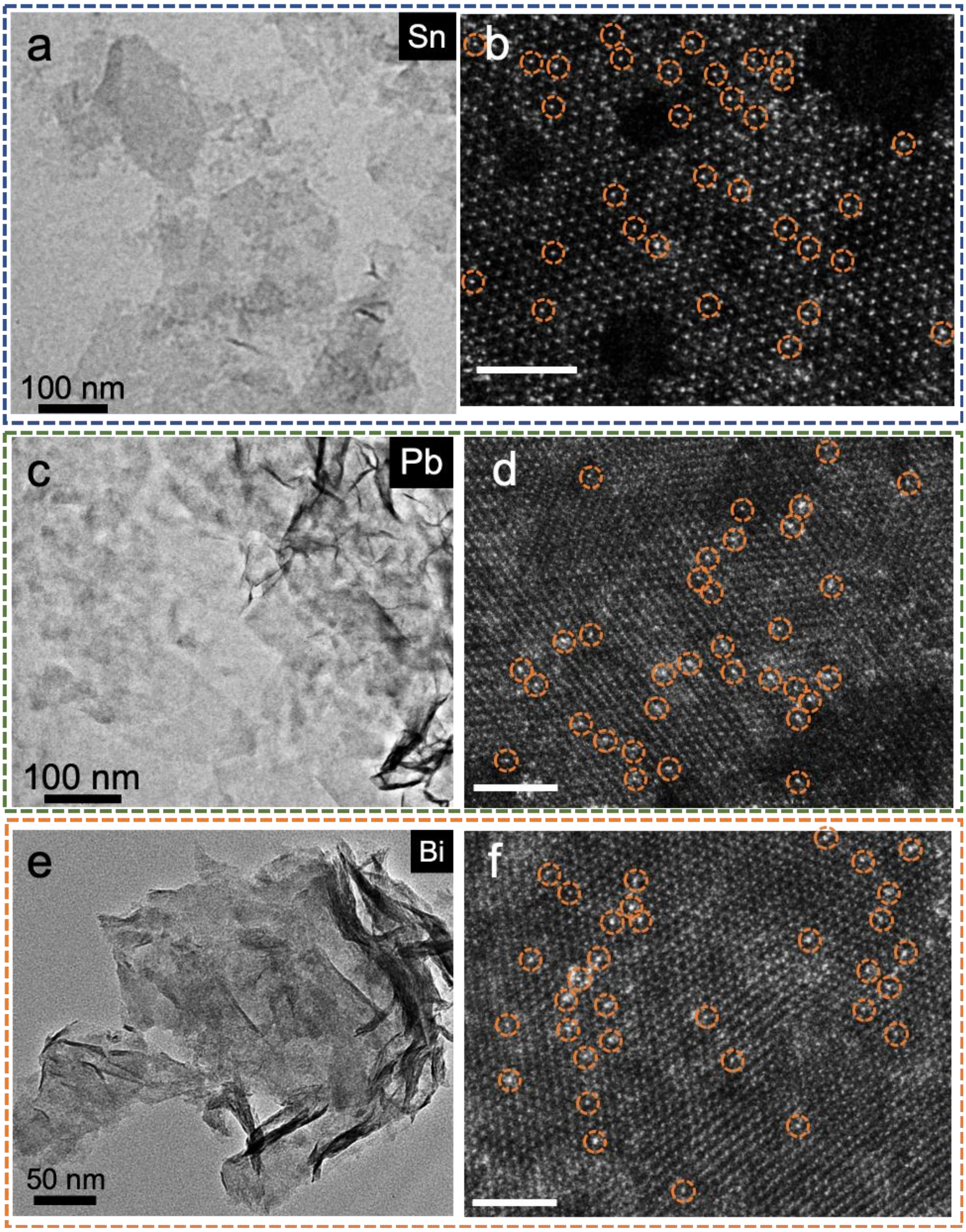

Supplementary Figure 10. (a, c, e) Conventional TEM images of freshly prepared Sn-SAs/MoS_2, Pb-SAs/MoS_2, and Bi-SAs/MoS_2 samples. (b, d, f) Magnified HAADF-STEM images of Sn-SAs/MoS_2, Pb-SAs/MoS_2, and Bi-SAs/MoS_2 samples (scale bar: 2 nm).

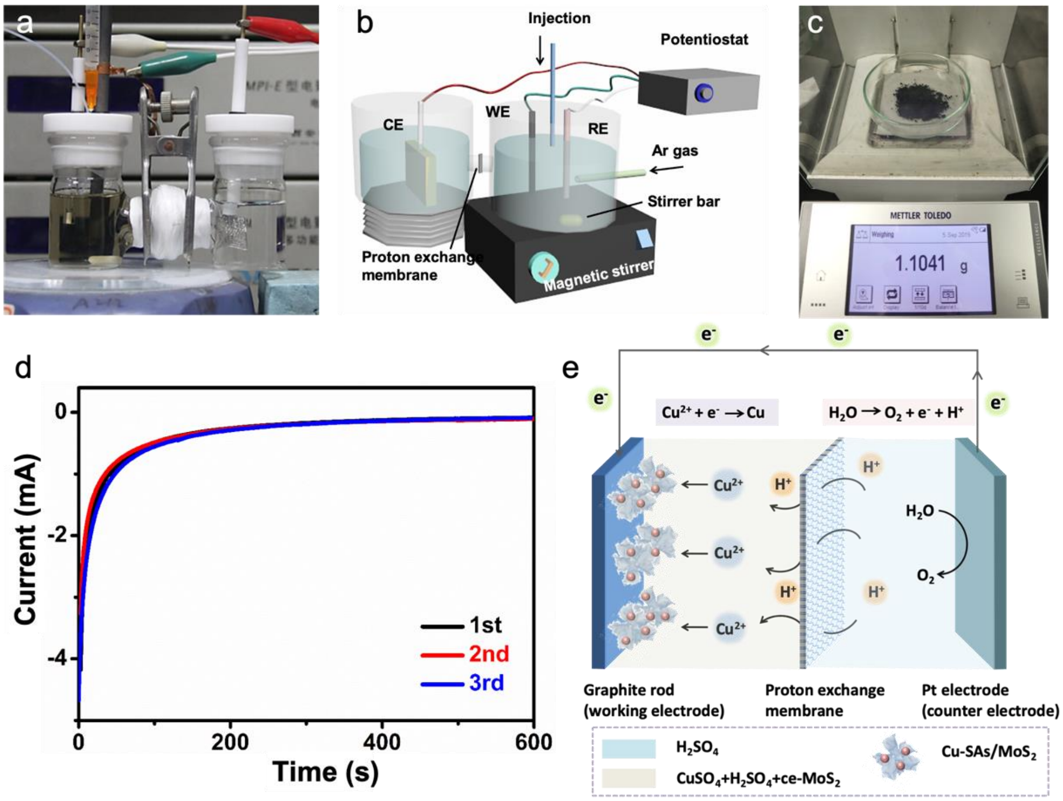

Supplementary Figure 11. Large-scale synthesis of Pt-SAs/MoS$_2$. (a) A photograph of the electrochemical apparatus (the synthetic procedure is described in the method section). (b) A 3D scheme of the cell showing the injector and different electrodes. (c) Photograph of the Pt-SAs/MoS$_2$ catalyst; the yield fulfills the requirement for large-scale production. (d) Chronoamperometry curves of the UPD of Cu in an Ar-saturated 0.1 M H_2SO_4 solution containing 2 mM $CuSO_4$ and 300 mg of ce-MoS$_2$ powder from three independent batches. The coincidence of the three independent experiments indicates the repeatability of this method. (e) Mechanism of the UPD of Cu on ce-MoS$_2$ nanosheets using the designed apparatus. Under vigorous stirring, the ce-MoS$_2$ nanosheets are underpotentially deposited with Cu atoms at the cathode ($Cu^{2+} + 2e^- \rightarrow Cu$) as they continuously collide with the graphite rod (working electrode). H_2O is oxidized at the anode (Pt wire electrode) to produce H^+ and O_2 gas ($2H_2O \rightarrow 4H^+ + O_2 + 4e^-$). The proton-exchange membrane acts as an electronic insulator and reactant barrier to prevent the O_2 gas produced at the anode from diffusing to the cathode; diffusion of dissolved platinum ions to the cathode is also avoided. H^+ ions cross through the membrane from the anode to the cathode to balance the pH between the two half-reaction cells. Electrons flow through the external circuit to the cathode to continuously reduce the Cu^{2+} ions.

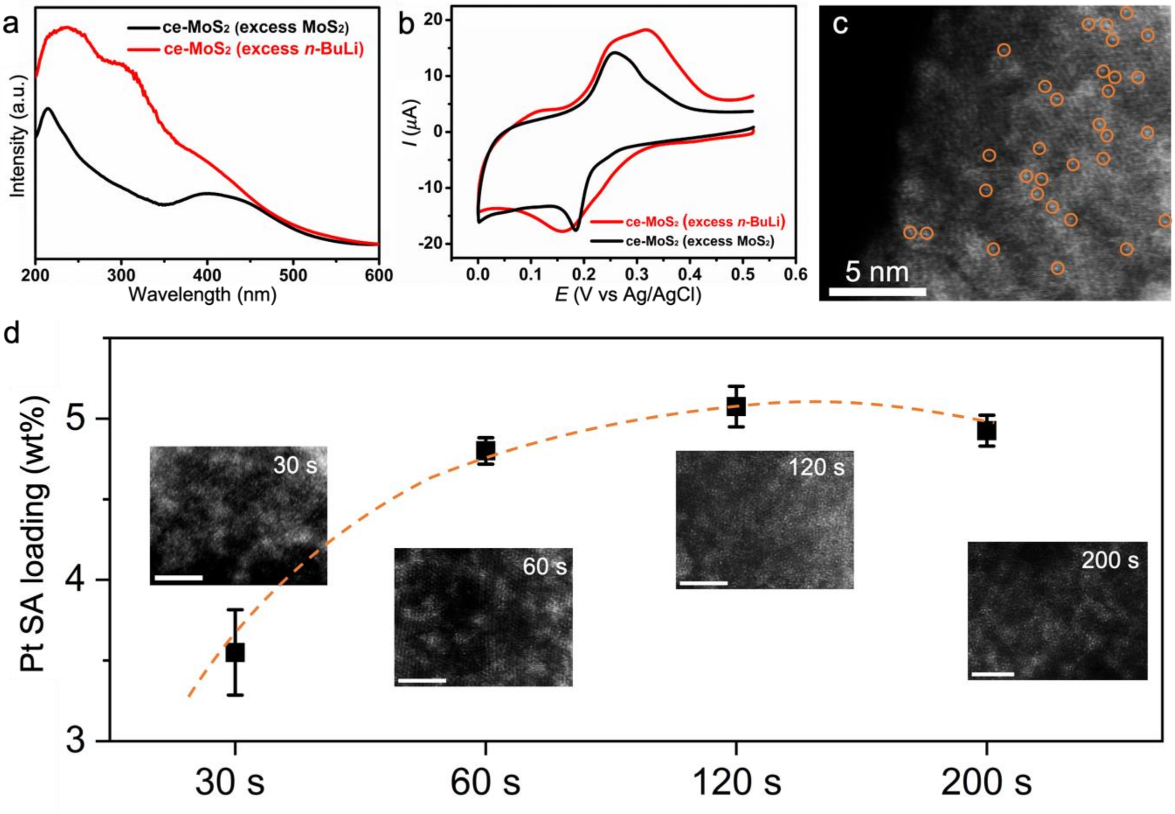

Supplementary Figure 12. Adjusting the loading of single-atom metal by controlling the number of active sites for Cu UPD or by controlling Cu UPD time. (a) UV-visible spectra of ce-MoS_2 with excess MoS_2 and excess *n*-BuLi. (b) CVs of ce-MoS_2 with excess MoS_2 and excess *n*-BuLi in an Ar-saturated 0.1 M H_2SO_4 solution containing 2 mM $CuSO_4$ at a scan rate of 20 mV s^{-1}. (c) Magnified HAADF-STEM image of the single Pt atoms on the ce-MoS_2 (excess MoS_2) nanosheets. The loading of Pt was 1.8 wt%, as measured by ICP-OES. (d) The Pt SA loading of Pt-SAs/MoS_2 with different Cu UPD times, as measured by ICP-OES, in an Ar-saturated 0.1 M H_2SO_4 solution containing 2 mM $CuSO_4$. As the deposition time increased in the first ca. 60 s, the loading of the single-atom Pt increased rapidly. After ca. 60 s, the loading increased very slightly, and finally remained unchanged, indicative of the saturated deposition. Error bars represent the standard deviation from three independent batches. The insets show the corresponding representative aberration-corrected HAADF-STEM images of Pt-SAs/MoS_2 with different UPD times (30 s; 60 s; 120 s; 200 s; scale bars: 5 nm). Similarly, as the deposition time increased, the number of atomically dispersed brightest white dots also increased, indicating that the loading amount of Pt single atoms could be precisely controlled by adjusting the deposition time.

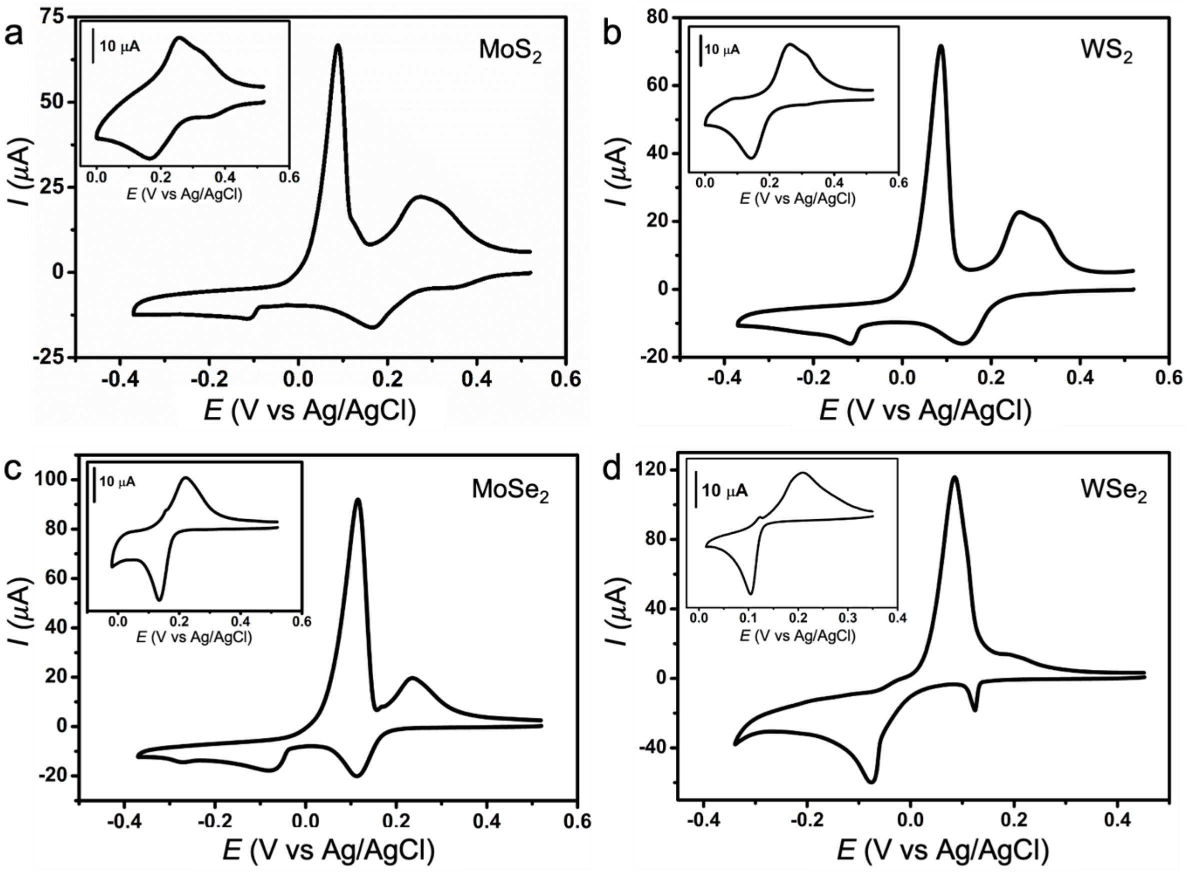

Supplementary Figure 13. Representative CVs of Cu UPD on various TMDs. CVs for ce-MoS_2 (a), ce-WS_2 (b), ce-$MoSe_2$ (c), and ce-WSe_2 (d) modified GCE electrodes in an Ar-saturated 0.1 M H_2SO_4 solution containing 2 mM $CuSO_4$ at a scan rate of 20 mV s^{-1}. Inset: the CVs restricted to the Cu UPD regions.

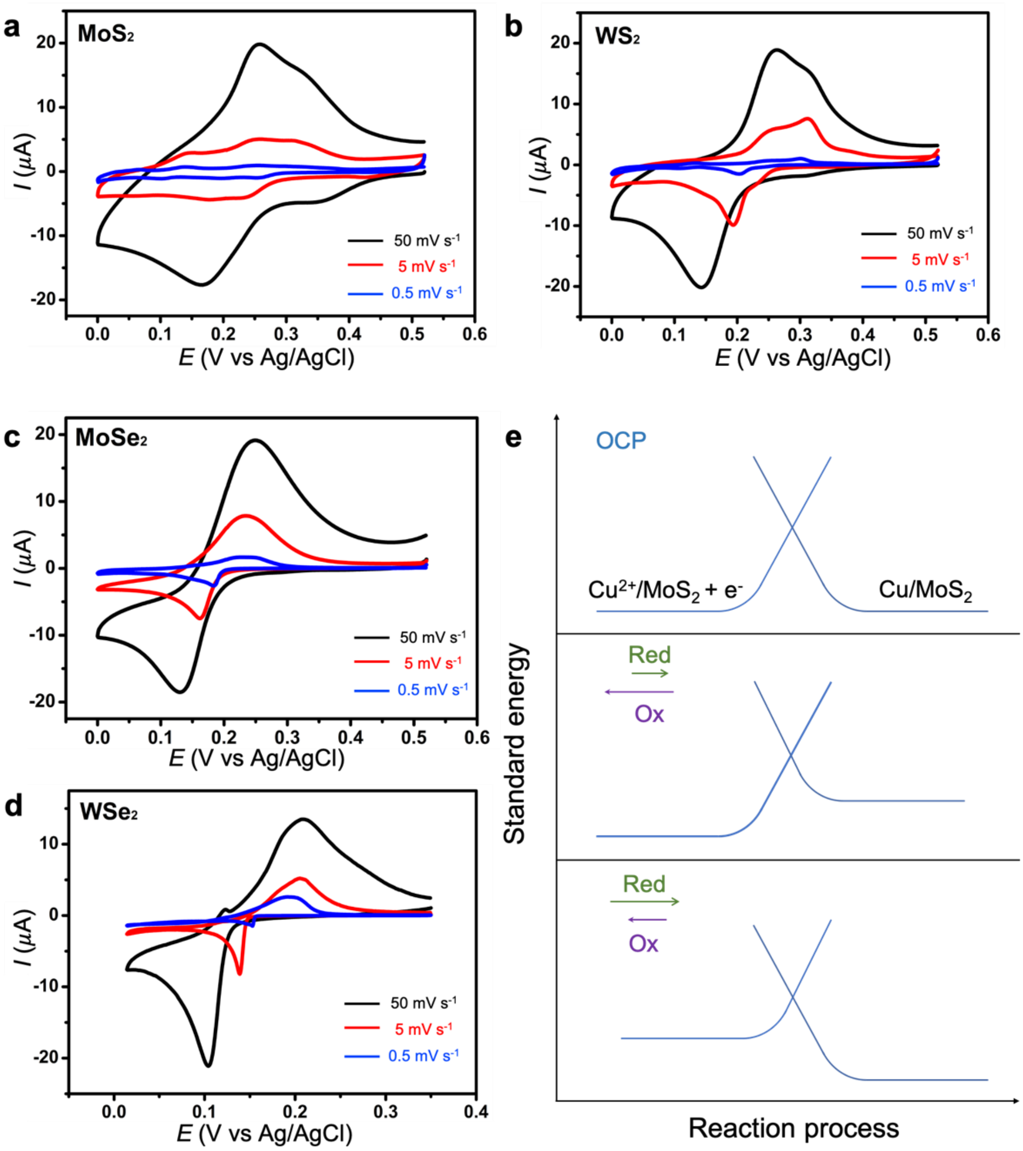

Supplementary Figure 14. Reversibility of Cu UPD on various TMDs. CVs of various TMD materials: (a) MoS_2, (b) WS_2, (c) $MoSe_2$, and (d) WSe_2 in an Ar-saturated 0.1 M H_2SO_4 solution containing 2 mM $CuSO_4$ at different scan rates. As the scan rate decreased (from 50 to 5 to 0.5 mV s^{-1}), a higher degree of voltammetric reversibility was apparent, as illustrated by the smaller reductive and oxidative peak separations, because the UPD current in the TMD materials take more time to respond to the applied potential. The scan rate that is sufficiently slow to make the anodic and cathodic currents totally symmetric is beyond the sensitivity of the electrochemical device. (e) Schematic illustration of standard free energy changes during the reversible UPD process ($Cu^{2+}/MoS_2 + e^- \leftrightarrow Cu/MoS_2$), (top) at the open-circuit potential, (middle) at a more positive potential than the UPD value, and (bottom) at a more negative potential than the UPD value (Supplementary Note 4).

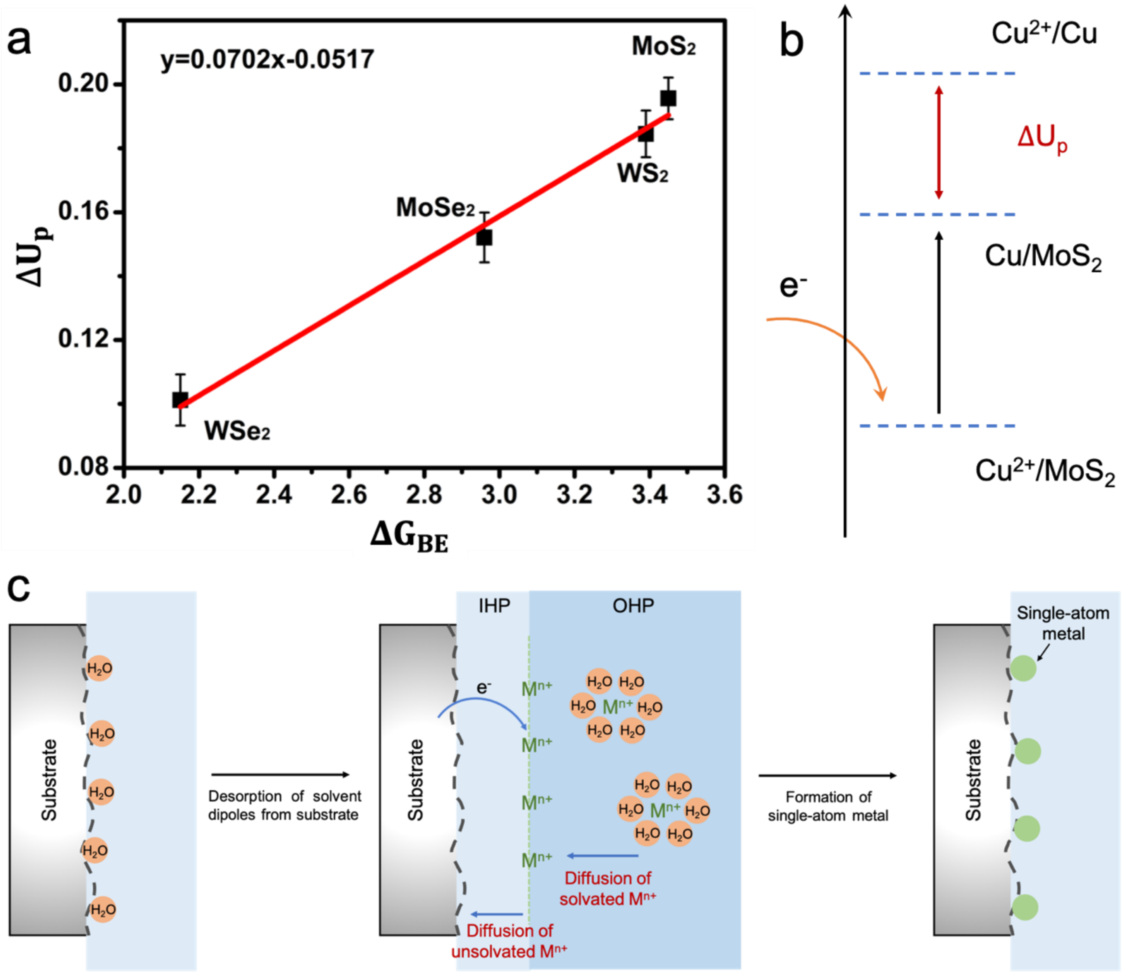

Supplementary Figure 15. Correlation between underpotential shift and binding energy. (a) Underpotential shift ΔU_{p} between bulk deposition and UPD as a function of single-atom–support bonding (ΔG_{BE}). The data are taken from Supplementary Table 6, and error bars represent the standard deviation from four independent batches. Red line is the linear fitting of the data. (b) Schematic diagram illustrating the redox potential of copper species in electrochemistry. The black arrows indicate the directions of potential change. The red arrow indicates the difference in potential between the UPD and the bulk deposition of Cu (for a further mechanistic discussion, see Supplementary Note 4). (c) Schematic representation of site-specific UPD process constituting the adsorption of metal (M) upon substrate. IHP represents the inner Helmholtz plane. OHP represents the outer Helmholtz plane.

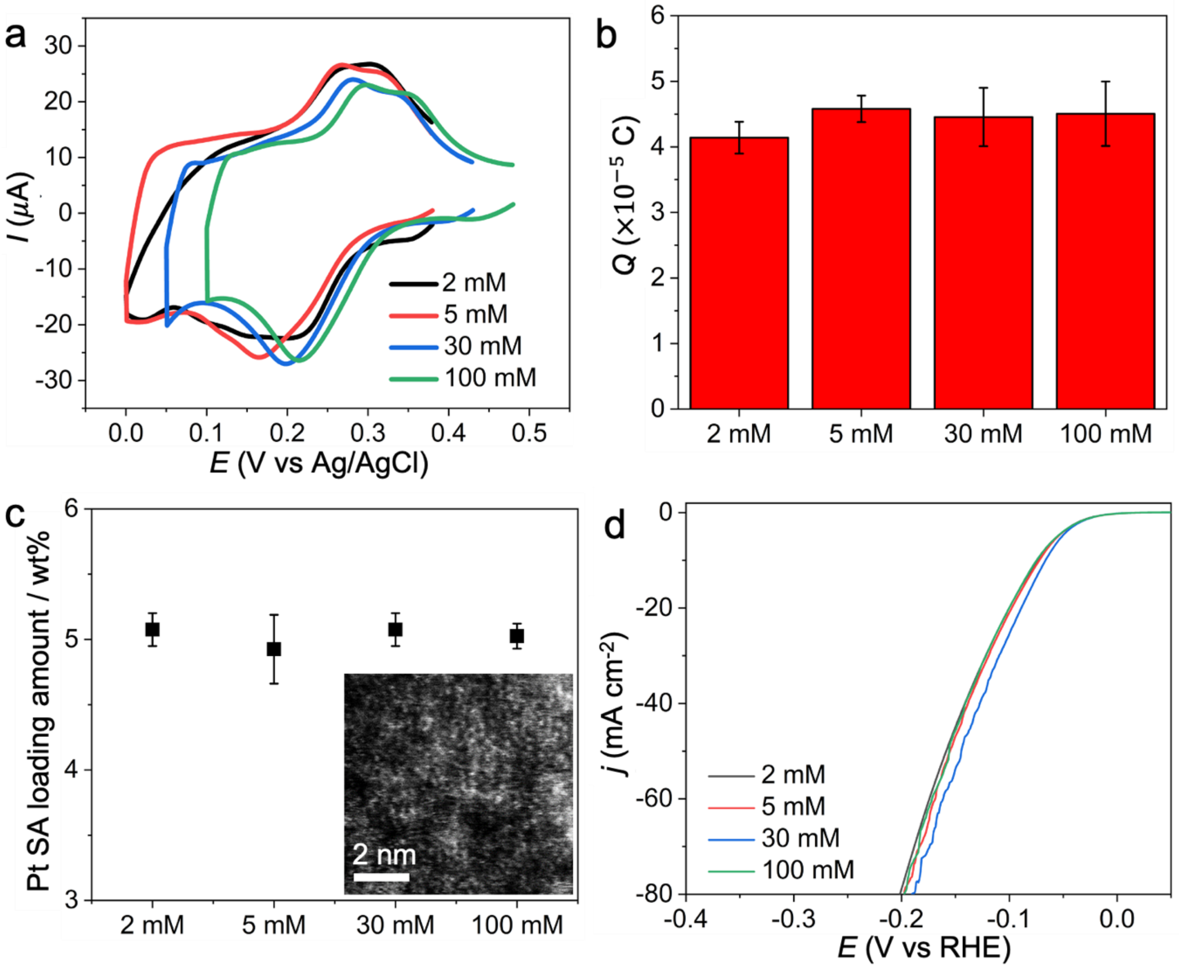

Supplementary Figure 16. The influence of precursor concentration on Cu UPD. (a) CV curves of ce-MoS_2 in an Ar-saturated 0.1 M H_2SO_4 solution containing different concentrations (2 mM, 5 mM, 30 mM, and 100 mM) of $CuSO_4$. (b) The corresponding charge quantity derived from the cathodic peak of Cu UPD in (a). (c) The Pt SA loading of Pt-SAs/MoS_2 synthesized with different concentrations of $CuSO_4$ for Cu UPD. Error bars represent the standard deviation from four independent batches. Inset shows the aberration-corrected HAADF-STEM image of Pt-SAs/MoS_2 synthesized with 100 mM $CuSO_4$ for Cu UPD. (d) HER polarization curves of Pt-SAs/MoS_2 synthesized with different concentrations of $CuSO_4$ for Cu UPD. The concentration of metal precursor for UPD has no significant influence on the single-atom growth, the final loading of single-atom Pt, and the catalytic activity. In particular, the excessively high concentration (100 mM) of metal precursor did not lead to the formation of single-atom aggregation or nanoparticles (some examples of concentration previously reported are summarized in Supplementary Table 8, typically <10 mM).

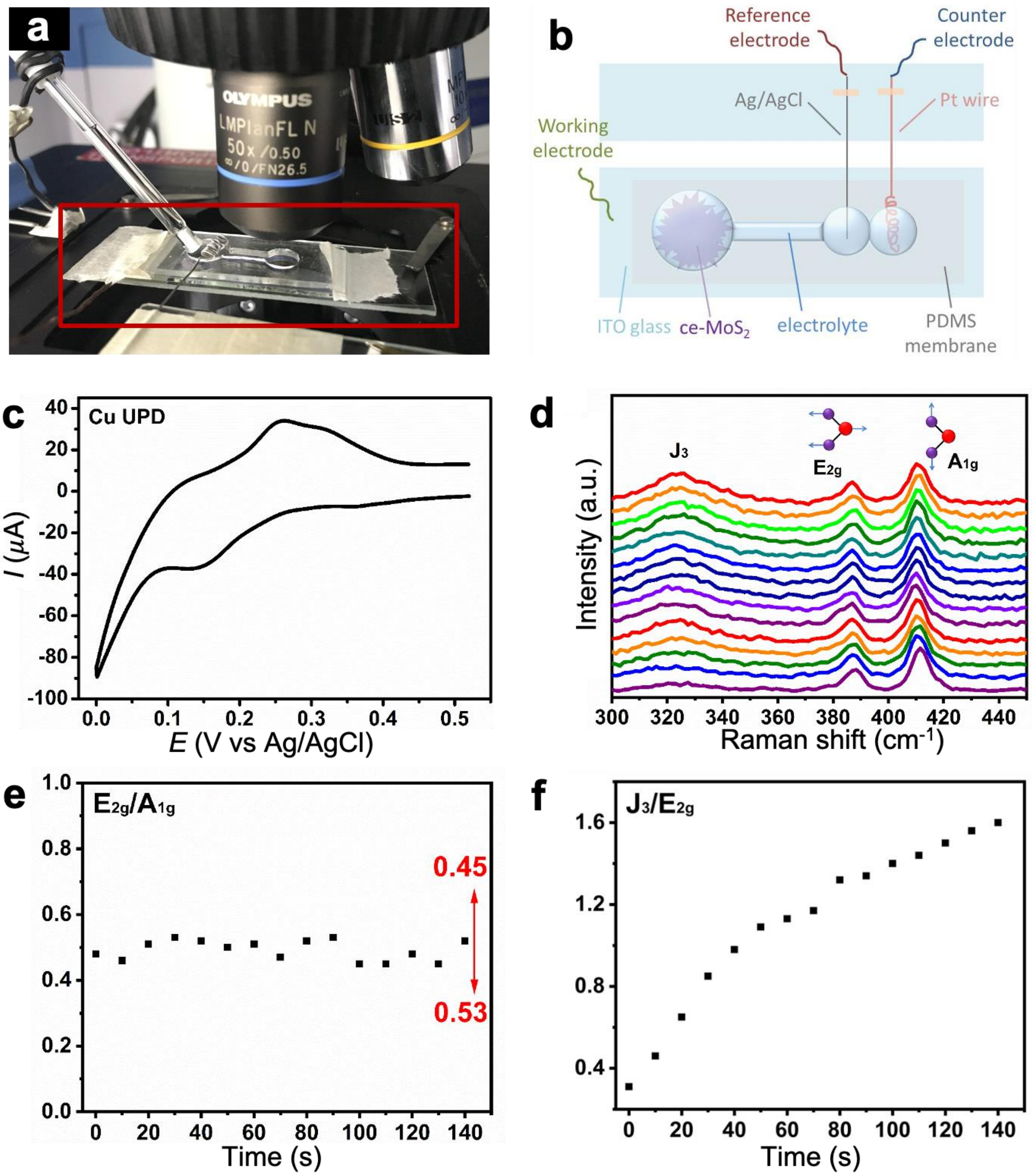

Supplementary Figure 17. Cu UPD process investigated by operando Raman spectroscopy and electrochemical techniques. (a) A photograph of the in-house-built electrochemical cell. (b) Vertical schematic view of the electrochemical cell. (c) A typical CV curve of ce-MoS_2-coated ITO glass in an Ar-saturated 0.1 M H_2SO_4 solution containing 2 mM $CuSO_4$ at a scan rate of 20 mV s^{-1}. (d) Raman spectra of ce-MoS_2 nanosheets under an applied potential of 0.1 V (vs Ag/AgCl) in an Ar-saturated 0.1 M H_2SO_4 solution containing 2 mM $CuSO_4$ with an acquisition time of 15 s for each spectrum. The prominent Raman bands at 387

and 411 cm^{-1} are assigned to the in-plane E_{2g} and out-of-plane A_{1g} modes, respectively (purple ball, S atoms; red balls, Mo atoms). The A_{1g} mode denotes the vibration for the out-of-plane lattice with two S atoms directly bound to Mo atoms moving in opposite directions, whereas the E_{2g} mode denotes the vibration for the in-plane lattice with the two S atoms collectively moving in the same direction opposed to the movement of Mo atom. An obvious blue shift for both in-plane E_{2g} mode and out-of-plane A_{1g} mode was observed, which suggests the n-doping polarity, and thus the increased electron density in MoS_2. (e, f) Statistical analysis of the relative intensity in E_{2g} and A_{1g} (e) or J_3 and E_{2g} (f) vibration modes induced by the UPD of Cu. The y-axes in (e) and (f) represent the ratio data. The relative intensity in E_{2g} and A_{1g} remains steady with acceptable fluctuation between 0.45 and 0.53, indicating the preservation of 1T-phase MoS_2. The relative intensity in J_3 and E_{2g} gradually increases from 0.31 to 1.60. Notably, the J_3 peak became stronger and downshifted from 328 to 324 cm^{-1} with the functionalization of Cu adatoms on the nanosheets.

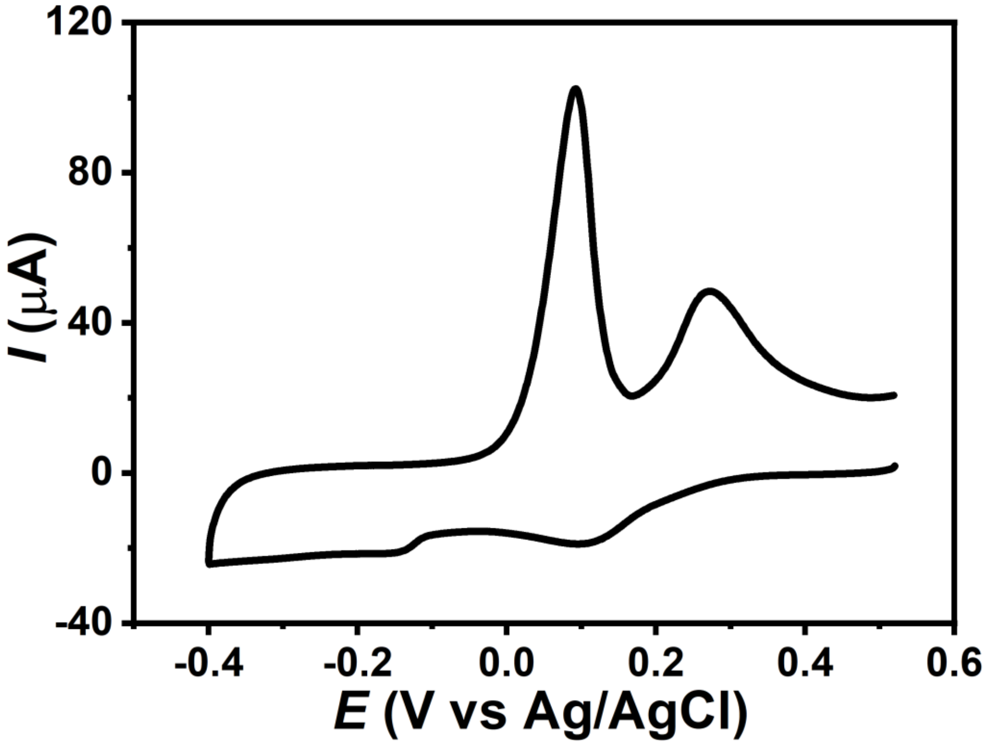

Supplementary Figure 18. CV of the I_2-treated ce-MoS_2-modified electrode in an Ar-saturated 0.1 M H_2SO_4 solution containing 2 mM $CuSO_4$ at a scan rate of 20 mV s^{-1}. We performed the control experiment by treating the ce-MoS_2 nanosheets with iodine, in which the residual charge was suppressed by mild oxidation. This result indicates that the I_2-treated ce-MoS_2, which has less surface charge, can still undergo UPD with Cu, which excludes the possibility of physisorption induced by weak noncovalent interactions.

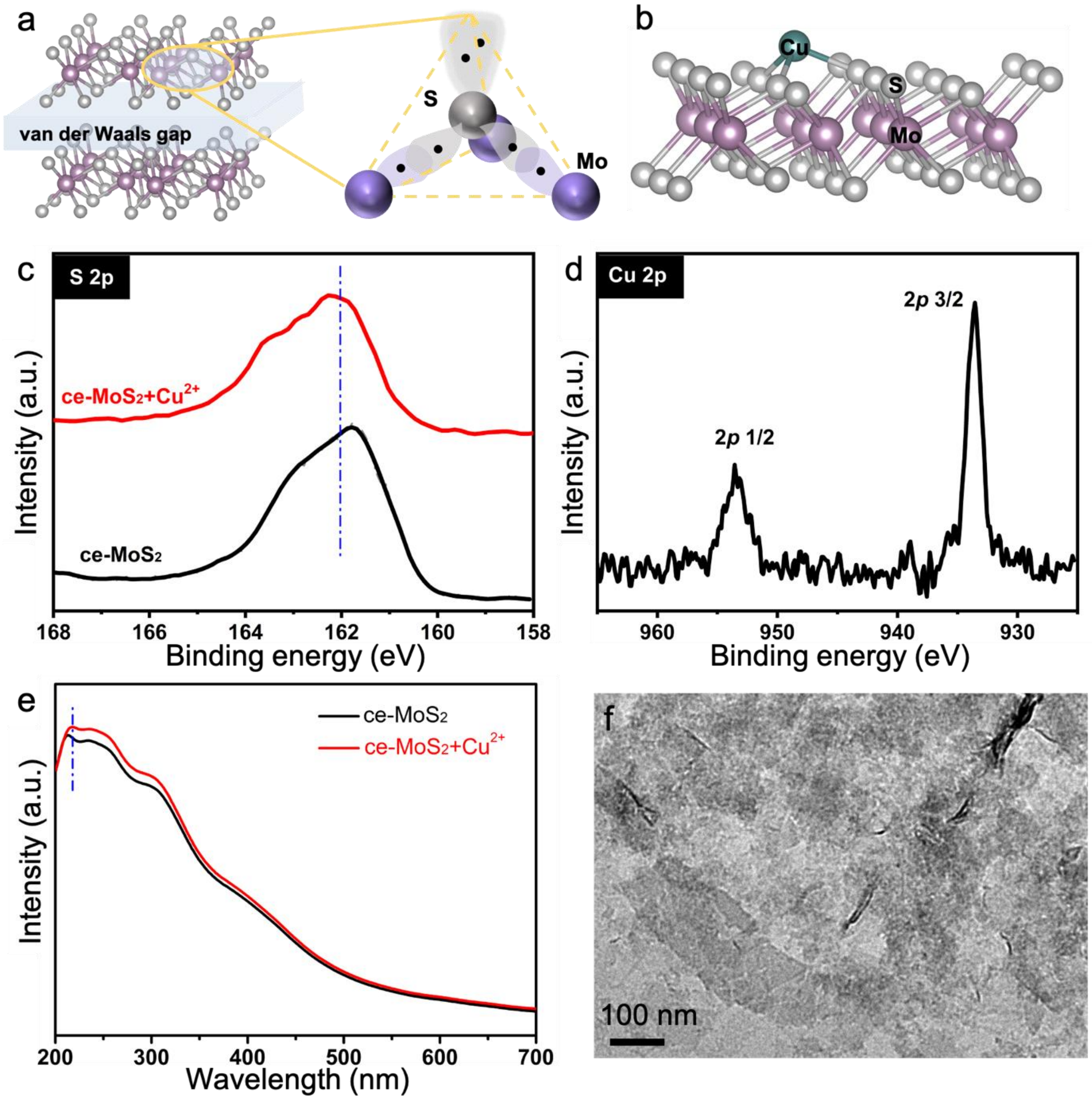

Supplementary Figure 19. Characterizations of Cu^{2+} ions adsorbed on ce-MoS_2. (a) Crystal structure model of double-layered MoS_2. Under the molecular orbital approximation, S atoms have an sp^3 hybridization configuration and the lone pair electrons on S are oriented into the lattice plane. (b) Structural model of Cu-SAs/MoS_2. XPS S 2p (c) and Cu 2p (d) spectra of the ce-MoS_2 nanosheets with adsorption of Cu^{2+} ions. (e) UV-visible spectra of the ce-MoS_2 nanosheets before and after adsorption of Cu^{2+} ions. (f) TEM image of the ce-MoS_2 nanosheets after adsorption of Cu^{2+} ions.

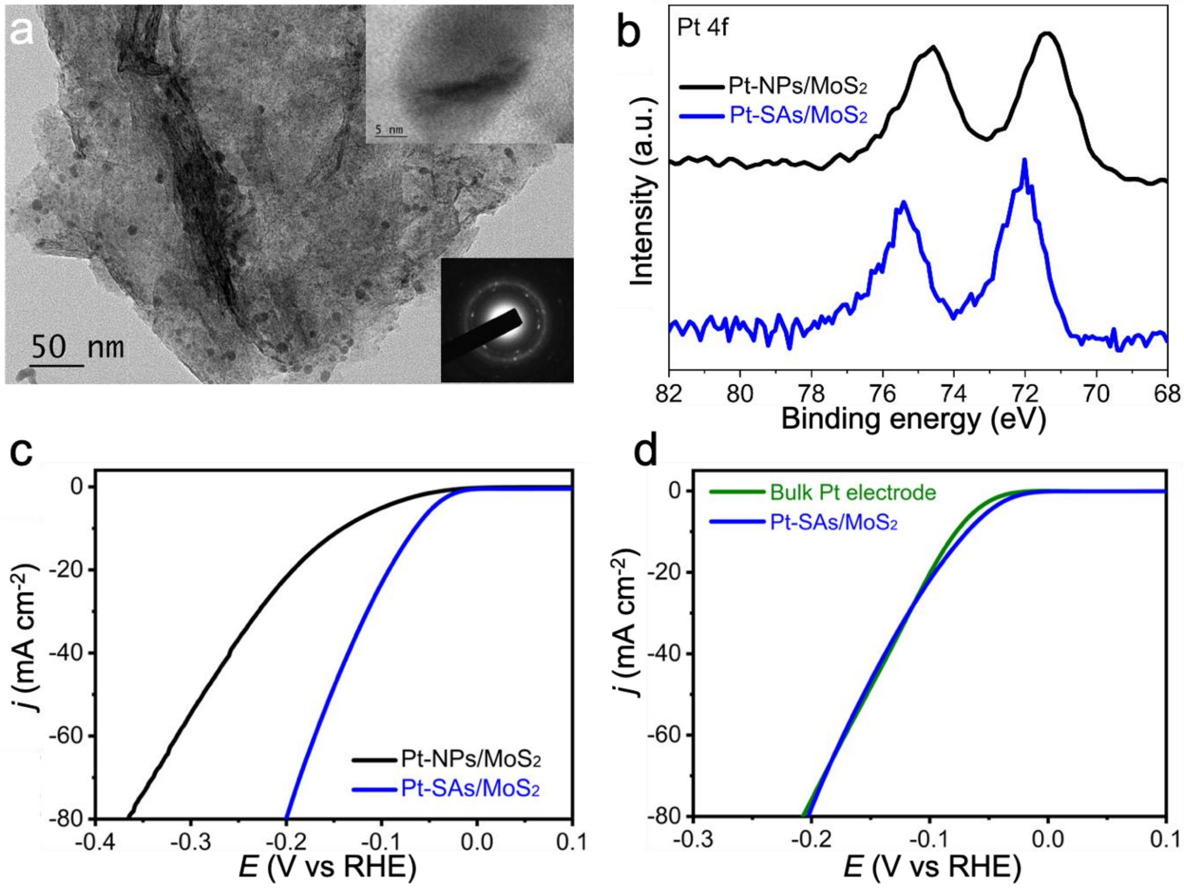

Supplementary Figure 20. Comparison of HER activity with a bulk Pt electrode and Pt-NPs/MoS_2. (a) A TEM image of Pt-NPs/MoS_2 (scale bar: 50 nm). The upper-right inset shows the high-resolution TEM of Pt-NPs/MoS_2 (scale bar: 5 nm). The lower-right inset shows the the corresponding SAED pattern of Pt-NPs/MoS_2. (b) Pt 4f XPS spectra of Pt-NPs/MoS_2 and Pt-SAs/MoS_2. (c) HER polarization curves of Pt-NPs/MoS_2 and Pt-SAs/MoS_2 in 0.5 M H_2SO_4 solution at a scan rate of 20 mV s^{-1}. As shown, Pt-NPs/MoS_2 exhibits decreased electrocatalytic HER activity, with an overpotential of 144 mV required to achieve 10 mA cm^{-2}, whereas an overpotential of only 59 mV is needed for Pt-SAs/MoS_2 to achieve the same activity. (d) HER polarization curves for the bulk Pt electrode and Pt-SAs/MoS_2 in an Ar-saturated 0.5 M H_2SO_4 solution at a scan rate of 20 mV s^{-1}.

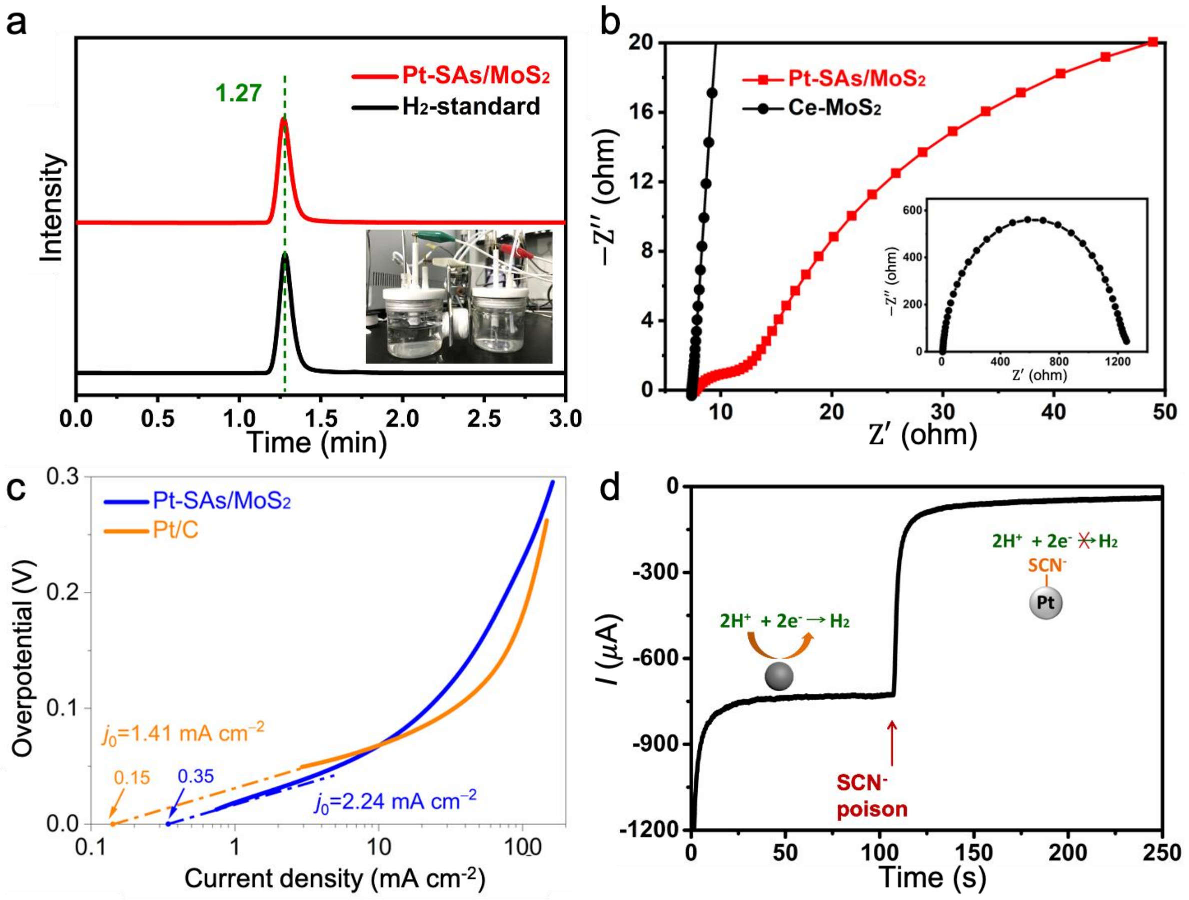

Supplementary Figure 21. Electrochemical HER performance. (a) gas chromatography (GC) of products catalyzed by Pt-SAs/MoS_2 (red line) in 0.5 M H_2SO_4; H_2-standard (black line): high-purity H_2 collected from the gas cylinder. The product catalyzed by Pt-SAs/MoS_2 during HER was detected using GC. Prior to gas collection, the electrolyte was thoroughly bubbled with Ar for 1 h. The product catalyzed by Pt-SAs/MoS_2 displays the same GC peak position as that of the H_2 standard, with a retention time of 1.27 min. No byproduct, only H_2, was observed. Inset: electrochemical device used in the GC measurements. (b) Electrochemical impedance spectra of Pt-SAs/MoS_2 and ce-MoS_2 at frequencies ranging from 0.1 Hz to 100 kHz with an amplitude of 10 mV at the onset potentials of each electrocatalyst at which the current density was 0.5 mA cm^{-2} in a solution of Ar-saturated 0.5 M H_2SO_4. The charge transfer resistance (R_{ct}) of the Pt-SAs/MoS_2 and ce-MoS_2 catalysts are 4.86 and 1246 Ω, respectively, which indicates a much lower R_{ct} after the surface of ce-MoS_2 was modified with Pt single atoms. This result suggests a much faster electron transfer rate and favorable HER kinetics with Pt-SAs/MoS_2. (c) Investigation of exchange current density of Pt-SAs/MoS_2 and commercial Pt/C on the basis of Tafel plots. (d) Current–time curve of Pt-SAs/MoS_2 before

and after the injection of SCN^- ions at -0.30 V (vs Ag/AgCl) in 0.5 M H_2SO_4. It is well known that metal catalytic sites can be deactivated by SCN^- ions under acidic conditions[39,40]. Before the addition of SCN^- ions, the active sites in Pt SA are exposed to the reacting substrate. After the addition, the active sites are bound by SCN^- ions and are hence blocked for HER catalysis, which further dramatically decreases the HER current from 736 to 35 μA.

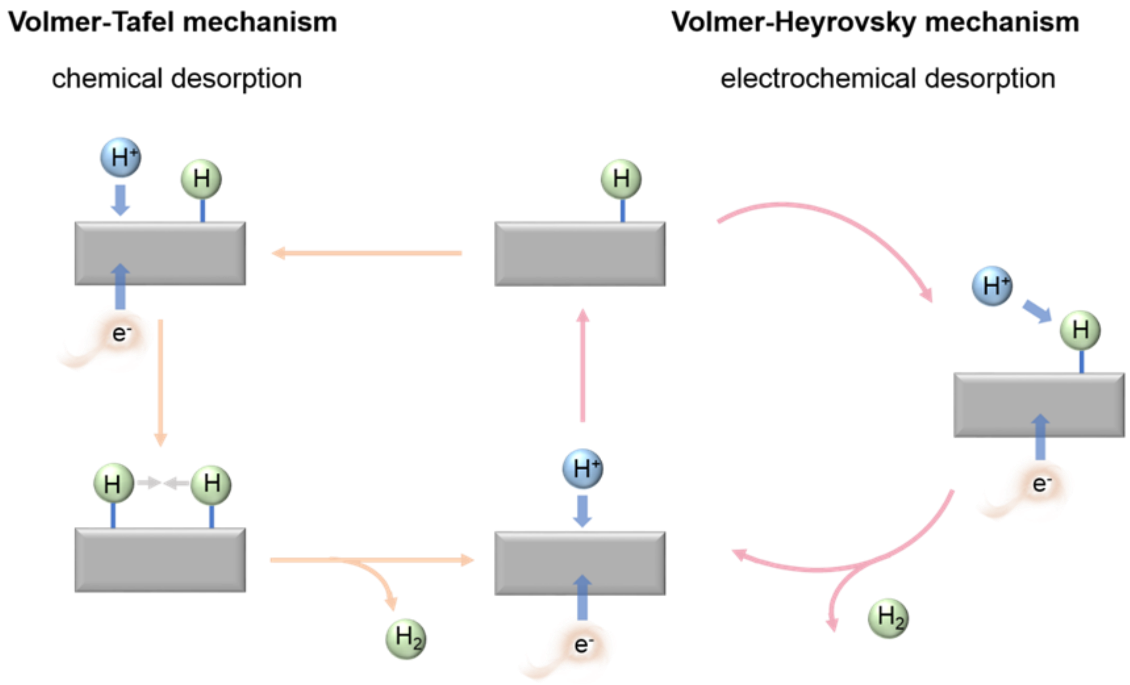

Supplementary Figure 22. Schematic representation of the HER mechanism under acidic conditions (for details, see Supplementary Note 6).

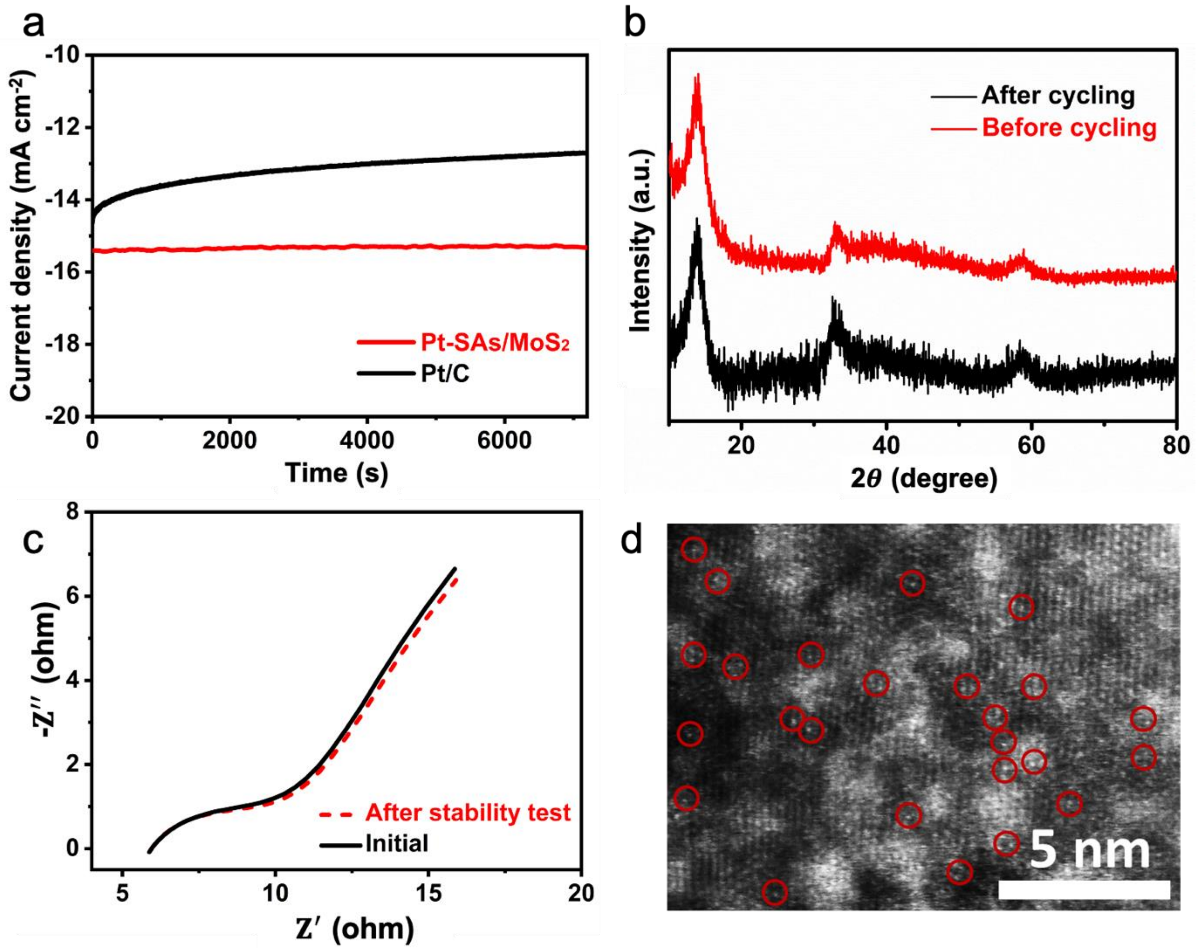

Supplementary Figure 23. HER stability of catalysts. (a) Chronoamperometry curves of Pt-SAs/MoS_2 and commercial Pt/C at a constant overpotential of 80 mV in an Ar-saturated 0.5 M H_2SO_4 solution for stability testing. (b) XRD patterns of Pt-SAs/MoS_2 before and after cycling. (c) EIS Nyquist plots of Pt-SAs/MoS_2 before and after cycling. No obvious change was observed, which indicates that Pt-SAs/MoS_2 is stable during HER catalysis. (d) HAADF-STEM image of Pt-SAs/MoS_2 after 1000 cycles. The XRD patterns remained almost unchanged after the stability test, indicating that the crystal structure is unaltered and that no obvious formation of Pt nanoparticles and clusters occurred. Moreover, from the HAADF-STEM image, bright spots of single Pt atoms were also observed, with no aggregation on Pt-SAs/MoS_2. No obvious change was apparent from the EIS spectra, strongly confirming that Pt-SAs/MoS_2 is relatively stable during the HER process.

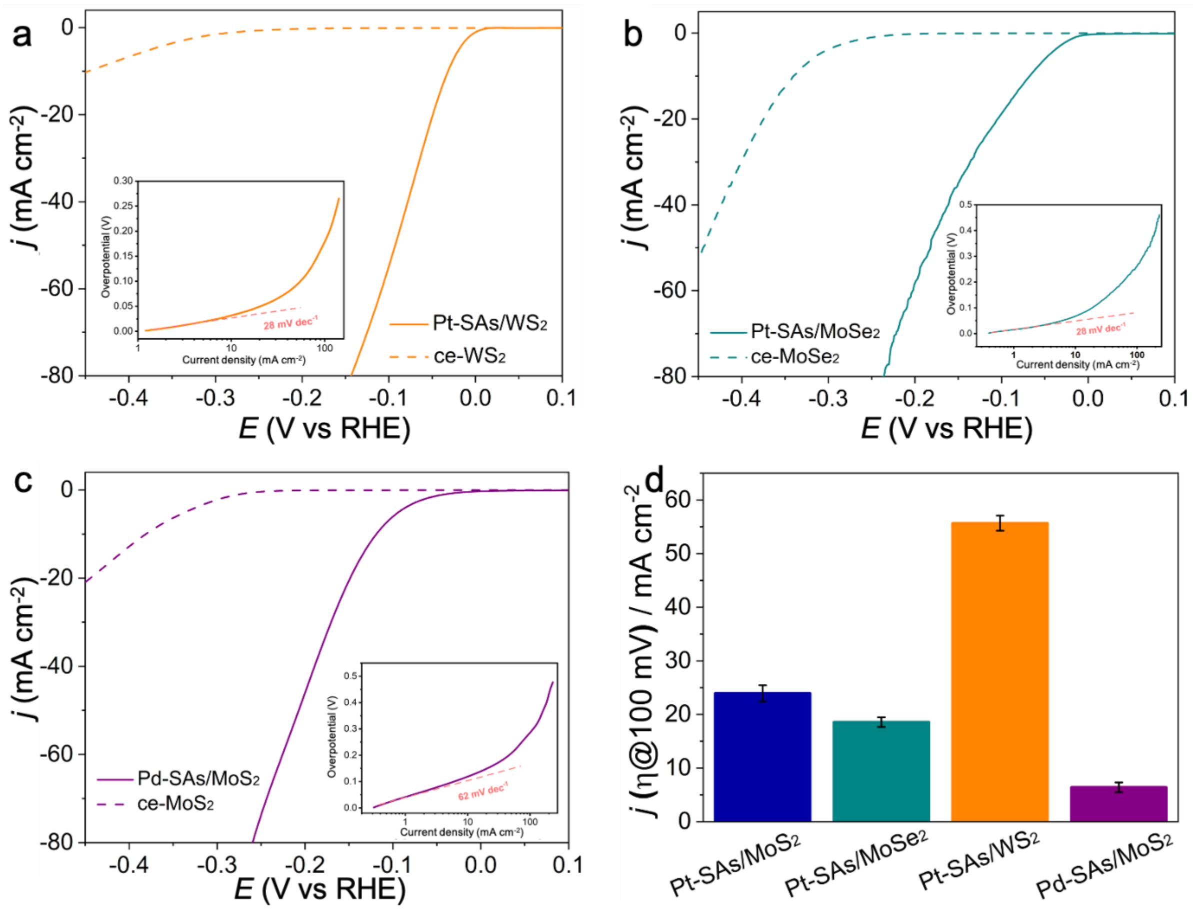

Supplementary Figure 24. HER activities of various single-atom catalysts. (a) Polarization curves for the HER catalyzed by ce-WS_2 and Pt-SAs/WS_2. Inset shows the Tafel plot for the HER catalyzed by Pt-SAs/WS_2. (b) Polarization curves for the HER catalyzed by ce-$MoSe_2$ and Pt-SAs/$MoSe_2$. Inset shows the Tafel plot for the HER catalyzed by Pt-SAs/$MoSe_2$. (c) Polarization curves for the HER catalyzed by ce-MoS_2 and Pd-SAs/MoS_2. Inset shows the Tafel plot for the HER catalyzed by Pd-SAs/MoS_2. (d) Current density of the indicated electrocatalysts obtained from HER polarization curves at an overpotential of 100 mV (error bars represent the standard deviation from six independent batches).

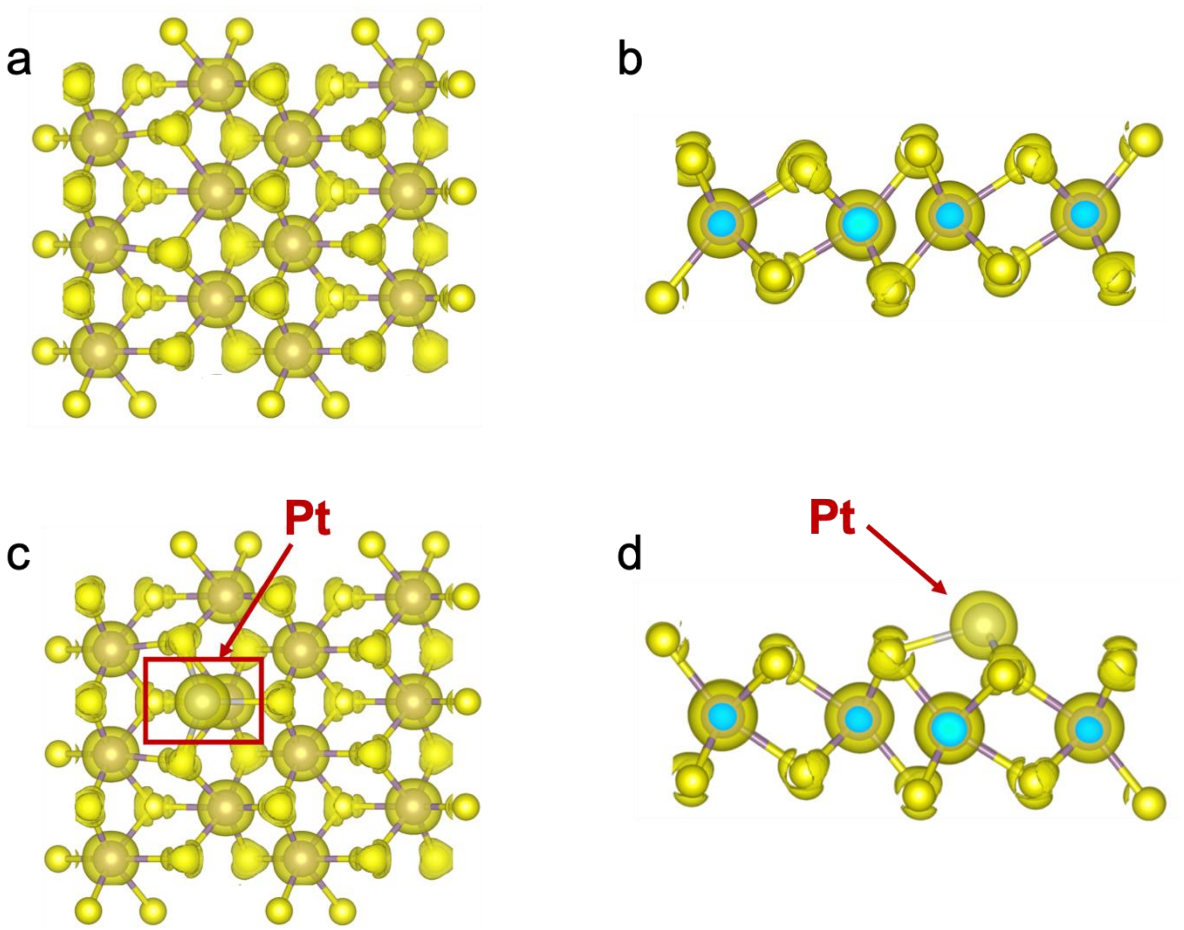

Supplementary Figure 25. Top view (a) and front view (b) of the electronic charge density distribution of MoS_2. Top view (c) and front view (d) of the electronic charge density distribution of Pt-SAs/MoS_2. The yellow and blue surfaces correspond to gain and loss of charge, respectively.

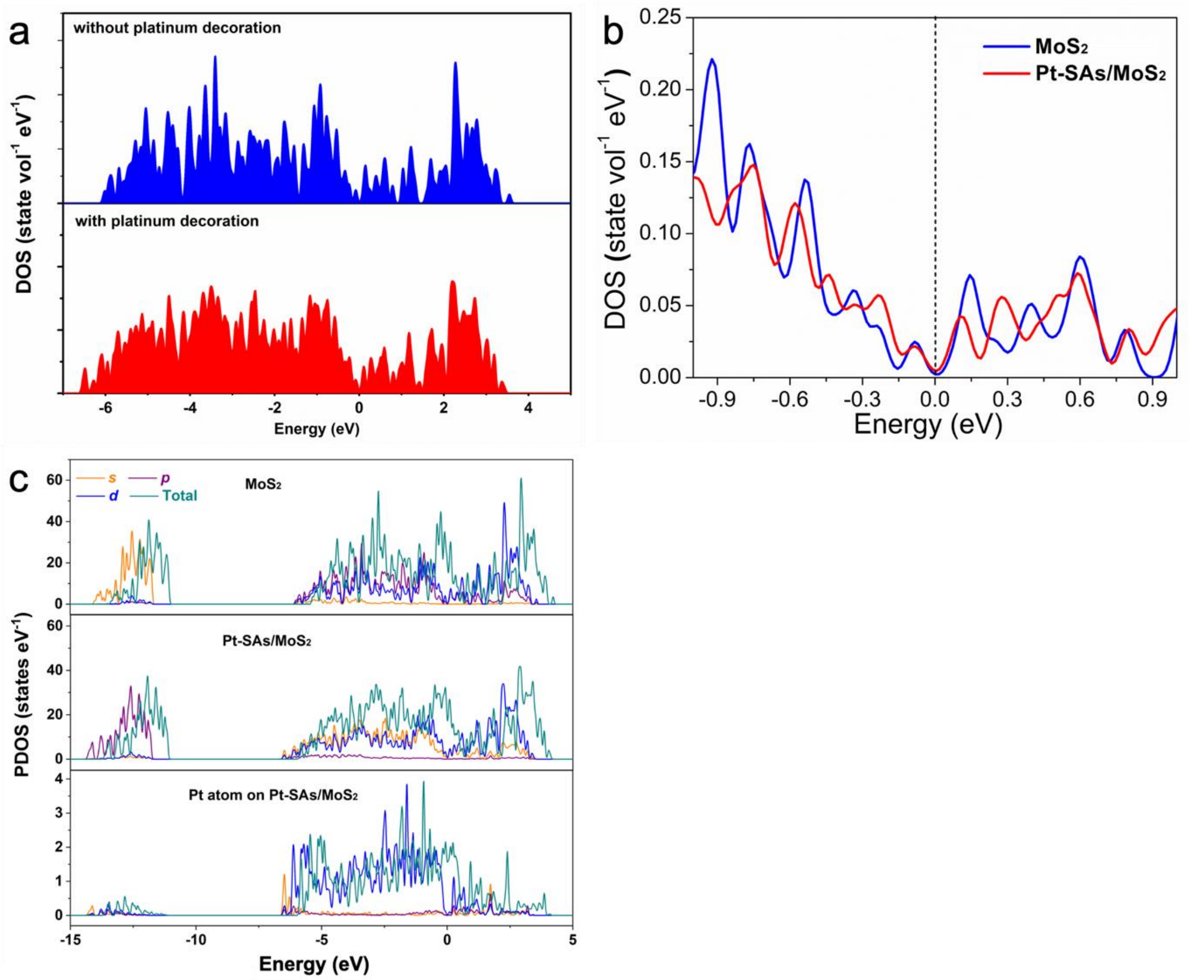

Supplementary Figure 26. DFT calculations on density of states. (a) Total density of states (TDOS) and (b) highlighted TDOS of MoS_2 and Pt-SAs/MoS_2. As illustrated by the TDOS, some new hybridized electronic states emerge in Pt-SAs/MoS_2 after Pt immobilization. This effect is due to the hybridization between Pt (5d orbitals) and the neighboring S atoms, in line with the EXAFS and XPS results. The enhanced DOS of Pt-SAs/MoS_2 near the Fermi level is mainly contributed by the Pt d orbitals. Both MoS_2 and Pt-SAs/MoS_2 have low DOS at the Fermi level and exhibit semi-metal properties. The immobilization of single-atom Pt on MoS_2 pushes the peak of DOS in MoS_2 towards the Fermi level, which is associated with promoted electron transfer, thus enhancing the conductivity of Pt-SAs/MoS_2. (c) Calculated PDOS of MoS_2 and Pt-SAs/MoS_2 with aligned Fermi levels from the aspect of molecular orbitals.

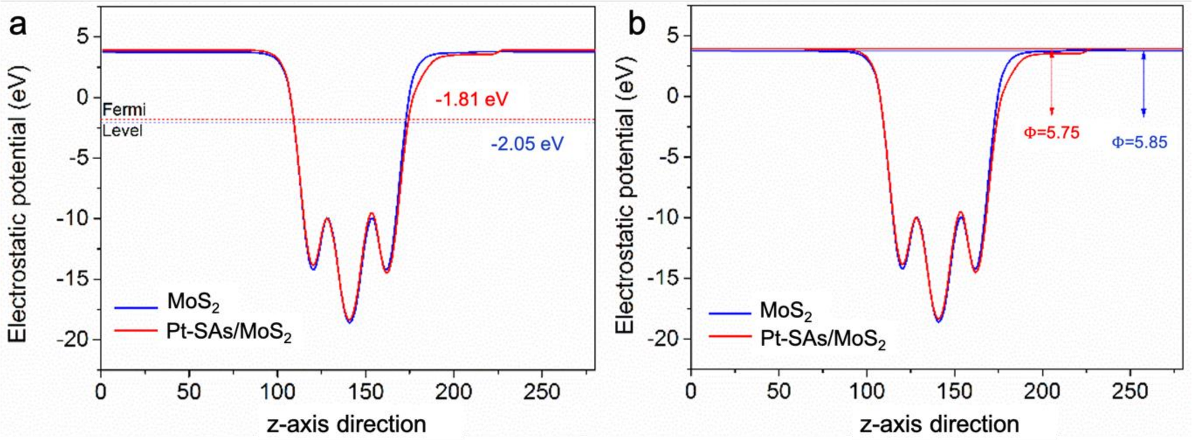

Supplementary Figure 27. DFT calculations on the (a) Fermi levels and (b) work functions of catalysts. The work functions were computed here as the energy difference between the electrostatic potential and the Fermi level. The calculated values of the work function are nearly 6 eV, which implies that both materials are capable of maintaining electron transfer and stability under experimental conditions. The electronic energy level of Pt-SAs/MoS_2 (-1.81 eV) is higher than that of the pure MoS_2 (-2.05 eV). Additionally, the work function of Pt-SAs/MoS_2 (5.75 eV) is lower than that of MoS_2 (5.82 eV), which means that Pt-SAs/MoS_2 has an enhanced ability to provide electrons.

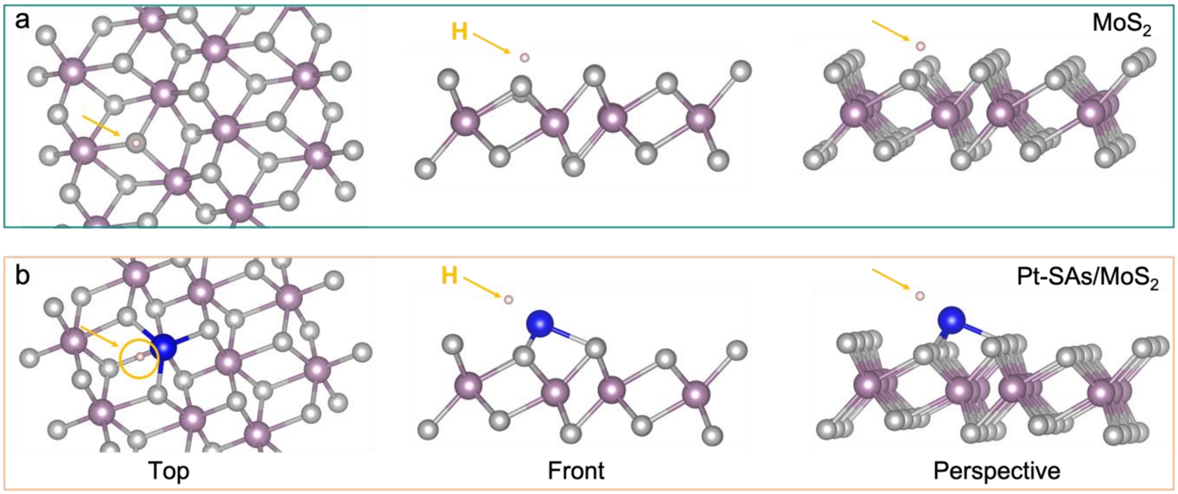

Supplementary Figure 28. (a) Top, front and perspective views of the DFT-calculated geometries for MoS_2 with an H atom absorbed on the S top site. (b) Top, front and perspective views of the DFT-calculated geometries for Pt-SAs/MoS_2 with an H atom absorbed on the Pt top site. The adsorption site of H is confirmed by comparing the energy of hydrogen adsorption at different positions on the catalyst surface after free geometry optimizations with an optimization tolerance level of 2.0×10^{-5} a.u. The atom color code is the same as in Supplementary Fig. 5.

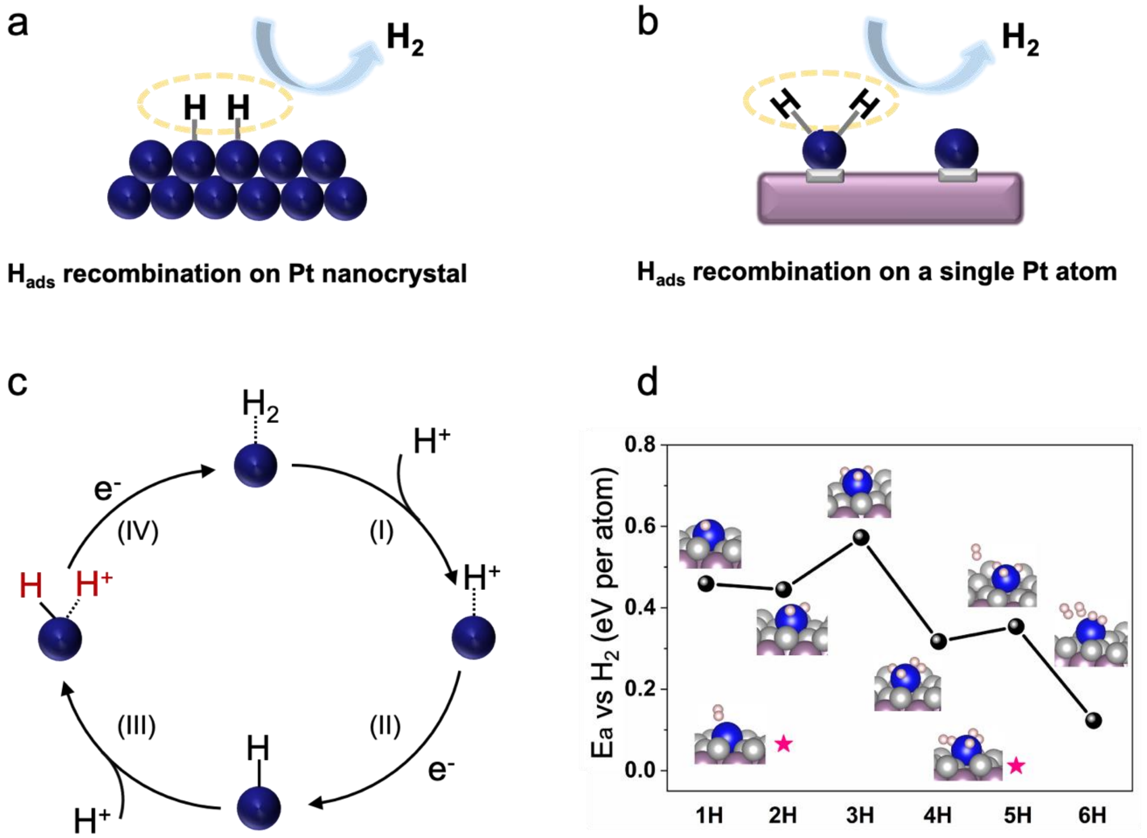

Supplementary Figure 29. The HER mechanism investigation of Pt-SAs/MoS_2. Proposed plausible HER mechanism of hydrogen recombination and desorption on commercial Pt/C (a) and Pt-SAs/MoS_2 (b and c). During the initial Volmer steps (Supplementary Fig. 29c, step I and II), adsorbed H^+ ions are chemically bonded to the Pt surface in the form of Pt–H bonds. However, during the following Tafel reaction, for traditional Pt nanocrystals, the two protons bind to two adjacent Pt atoms, combine, and generate a H_2 molecule (Supplementary Fig. 29a), whereas for Pt SAs, the two protons bind to a single Pt atom, combine and produce H_2 (Supplementary Fig. 29b; step III and IV in Supplementary Fig. 29c). More detailed description is shown in Supplementary Note 8. (d) Calculated adsorption energies of H atoms as a function of the H coverage (one to six H atoms) on a single Pt atom in Pt-SAs/MoS_2. Blue, gray, purple and pink spheres indicate Pt, S, Mo and H atoms, respectively. The black spheres represent the energy of the most stable adsorption structure per H loading. The red stars indicate the adsorption configurations with two and five H atoms, which form one and two H_2 dimers on a Pt atom, respectively. All the hydrogen adsorption sites were structurally optimized with the most stable geometries (for a further discussion, see Supplementary Note 8).

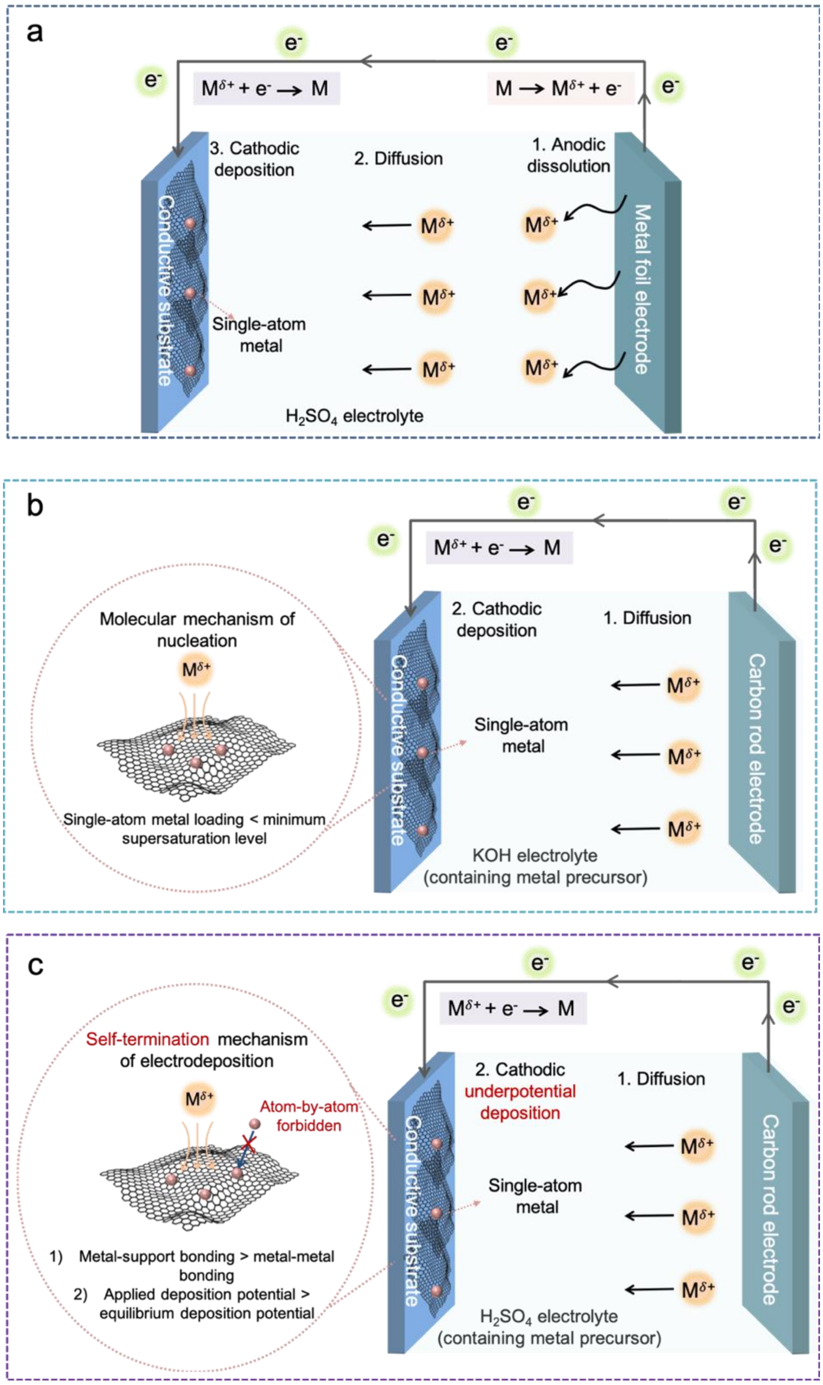

Supplementary Figure 30. Comparison between the previously reported electrodeposition method and SSED for ADMC synthesis. (a) Traditional single-atom electrodeposition mechanism[16-20]. (b) Cathodic/anodic electrodeposition (C, A-ED) mechanism of single-atom metals reported by Zeng's group[21] (taking cathodic deposition as an example). (c) Self-terminating growth mechanism of single-atom metals reported by our group (for a further discussion, see Supplementary Note 9 and Supplementary Table 10).

Supplementary Tables

Supplementary Table 1. Optimization of the loading concentration of ce-MoS_2 on a glassy carbon electrode.

Loading concentration *m* (mg mL^{-1})	Charge quantity *Q* (C)	*Q*/*m* ratio
1.0	1.243×10^{-4}	1.243×10^{-4}
0.5	8.436×10^{-5}	1.687×10^{-4}
0.25	5.759×10^{-5}	2.304×10^{-4}
*0.125	4.162×10^{-5}	3.330×10^{-4}
0.0625	1.480×10^{-5}	2.368×10^{-4}

*Charge (*Q*) corresponds to the UPD of Cu. The data in red row represent the optimal loading concentration of ce-MoS_2 on GCE for further synthesis of Pt SA, and electrocatalytic studies.

The excessive usage of ce-MoS_2 on the electrode could make it easily detach from GCE and lowers the utilization efficiency of the two-dimensional nanosheet material owing to interlayer stacking. Excessive stacking has a deleterious effect on the exposure of S sites for the Cu UPD process.

Supplementary Table 2. Zeta potential values of ce-MoS_2, iodine-treated ce-MoS_2, and Pt-SAs/MoS_2 (pH 7.4). The values are the averages of three measurements.

Entry	Zeta potential (mV)
ce-MoS_2	-49
Iodine-treated ce-MoS_2	-23
Pt-SAs/MoS_2	-27

The ce-MoS_2 nanosheets with large proportions of 1T phase are negatively charged (-49 mV, as indicated by zeta potential measurement), which allows them to have good colloidal stability and remain suspended (Supplementary Fig. 3)[41,42]. Heising *et al.* have previously calculated an excess charge of approximately 0.25 per ce-MoS_2 nanosheet[41]. The 2H phase of MoS_2 is not charged and hence cannot be stabilized in common solvents. After modification with metal atoms, the zeta potential of Pt-SAs/MoS_2 increases to -27 mV, which indicates that partial charges on the ce-MoS_2 nanosheets are neutralized by the attachment of single atoms.

To remove the residual charge on the ce-MoS_2 nanosheets, we carried out an iodine treatment experiment by immersing ce-MoS_2 in 0.15 M iodine in acetonitrile[27]. After this treatment, the zeta potential of ce-MoS_2 increased significantly (-23 mV) owing to the suppression of charge by mild oxidation[27]. We found that the UPD of Cu atoms is also possible on iodine-treated ce-MoS_2 (Supplementary Fig. 18), which strongly suggests that the S atoms with a lone electron pair are the active sites for the UPD process.

Supplementary Table 3. Summary of the representative single-atom metals prepared by various methods.

Catalyst	Metal	Loading	Timescale	Method	Reference
Pt-SA/MoS_2	Pt	5.1 wt%	min	SSED	This work
Pt-SAs/WS_2	Pt	4.1 wt%	min	SSED	This work
Pt-SAs/$MoSe_2$	Pt	4.7 wt%	min	SSED	This work
Pd-SAs/MoS_2	Pd	2.8 wt%	min	SSED	This work
Rh/ZnO	Rh	0.03 wt%	Hour	IWI	*Angew. Chem.* 2016, **128**, 16288
Pd/ZSM-5	Pd	0.01 wt%	Hour	IWI	*Angew. Chem.* 2016, **128**, 13639
Au/CeO_x	Au	0.3 wt%	Hour	IWI	*ACS Catal.* 2015, **5**, 6249
Pt/TiC	Pt	0.2 wt%	Hour	IWI	*ACS Catal.* 2017, **7**, 1301
Pt SA/WO_{3-x}	Pt	0.42 wt%	Hour	IWI	*Angew.Chem.* 2019, 131, 16184
Ru SAs@PN	Ru	0.33 wt%	Hour	IWI	*Angew. Chem. Int. Ed.* 2018, **57**, 9495
Pt_1/hNCNC	Pt	2.92 wt%	Hour	IWI	*Nat. Commun.* 2019, **10**, 1657
Pt/TiN	Pt	0.35 wt%	Hour	IWI	*Angew. Chem. Int. Ed.* 2016, **55**, 2058
Pt/TiO_2/MCM-41	Pt	0.5 wt%	Hour	IWI	*J. Am. Chem. Soc.* 2015, **137**, 3470
Pt@PCM	Pt	0.53 wt%	Hour	IWI	*Sci. Adv.* 2018, **4**, 6657
Co-NG	Co	0.57 atom%	Hour	IWI	*Nat. Commun.* 2015, **6**, 8668
Pt/HZSM-5/SiO_2	Pt	0.6 wt%	Hour	IWI	*Science* 2015, **350**, 189
M/meso_S-C	M=Pt, Pd, Ru, Ir, Rh	3-10 wt%	Hour	IWI	*Sci. Adv.* 2019, 5, 6322
Pt-PMA/AC	Pt	1 wt%	Hour	IWI	*Angew. Chem. Int. Ed.* 2016, **55**, 8319
Pt/θ-Al_2O_3	Pt	1 wt%	Hour	IWI	*J. Am. Chem. Soc.* 2013, **135**, 12634
Pt/NiS@Al2O3	Pt	2.8 wt%	Hour	IWI	*J. Mater. Chem. A* 2018, **6**, 11783
Pt/γ-Al_2O_3	Pt	1 wt%	Hour	IWI	*Science* 2009, **325**, 1670
A-Ni@DG	Ni	1.24 wt%	Hour	IWI	*Chem.* 2018, **4**, 1
Pt/MoS_2	Pt	1.7 wt%	Hour	IWI	*Energy Environ. Sci.* 2015, **8**, 1594
Pt_1@Fe-N-C	Pt	2.1 wt%	Hour	IWI	*Adv. Energy Mater.* 2018, **8**, 1701345
A-Ni-NSG	Ni	2.5 wt%	Hour	IWI	*Nat. Energy* 2018, **3**, 140
Pt_1@CeO_2	Pt	0.5-1.0 wt%	Hour	IWI	*ACS Catal.* 2018, 8, 4044
Pt/HSC	Pt	5 wt%	Hour	IWI	*Nat. Commun.* 2016, **7**, 10922
Pt/MoS_2	Pt	NA	Hour	IWI	*ACS Nano* 2017, **11**, 3392
Pt-PMA/AC	Pt	0.9 wt%	Hour	IWI	*J. Am. Chem. Soc.* 2019, **141**, 8185
(Fe,Co)/N-C	Fe/Co	0.93 / 1.17 wt %	Hour	IWI	*J. Am. Chem. Soc.* 2017, **139**, 17281
Ni_{SA}-MoS_2/CC	Ni	1.8 atom%	Hour	IWI	*Nano Energy* 2018, **53**, 458
MCM@MoS_2–Ni	Ni	2.7 wt%	Hour	IWI	*Adv. Funct. Mater.* 2018, **28**, 1807086
TM-CNT	TM=Fe , Pd, Co, Mn	0.1 atom%	Hour	IWI	*Nat. Commun.* 2019, **10**, 3997
Cu-CeO_2	Cu	4.05 atom%	Hour	IWI	*ACS Catal.* 2018, **8**, 7113
Pd/TiO_2	Pt	1.5 wt%	min	PRM	*Science* 2016, **352**, 797

Pt_1	Pt	NA	Hour	PRM	*Nat. Commun.* 2017, **8**, 1490
Pt_1/NPC	Pt	3.8 wt%	Hour	PRM	*ACS Catal*. 2018, **8**, 8450
AC Pt-NG/C	Pt	2.0 wt%	Hour	PRM	*ACS Catal*. 2019, **9**, 8213
Pt_1/MoO_{3-x}	Pt	2.0 wt%	Hour	PRM	*ChemCatChem* 2018, **10**, 946
Pt_1/NMC	Pt	2.54 wt%	Hour	PRM	*Chem. Sci.* 2019, **10**, 2830
Ir_1/FeO_x	Ir	0.01 wt%	Hour	CP	*J. Am. Chem. Soc.* 2013, **135**, 15314
Pt/FeO_x	Pt	0.17 wt%	Hour	CP	*Nat. Chem.* 2011, **3**, 634
$Ir_1/Pt_1/FeO_x$	Ir/Pt	0.22 / 0.17 wt%	Hour	CP	*J. Phys. Chem. C* 2014, **118**, 21945
$Pt/SnO_x/ZrO_x$	Pt	0.3 wt%	Hour	CP	*ChemCatChem* 2016, **8**, 1773
Pt/FeO_x	Pt	1.3 wt%	Hour	CP	*Adv. Mater.* 2014, **26**, 8147
Pt/MoS_2	Pt	7.5 wt%	Hour	SR	*Nat. Nanotechnol.* 2018, **13**, 411
$Pd/g\text{-}C_3N_4$	Pd	0.5 wt%	Hour	SR	*Angew. Chem. Int. Ed.* 2015, **54**, 11265
$Co\text{-}^sMoS_2$	Co	1.8 wt%	Hour	SR	*Nat. Chem.* 2017, **9**, 810
$Pt/g\text{-}C_3N_4$	Pt	0.16 wt%	Hour	SR	*Adv. Mater.* 2016, **28**, 2427
Pd/Cu-Pt	Cu/Pt	5 / 1.5 atom%	Hour	SR	*Angew. Chem. Int. Ed.* 2017, **56**, 16047
$Cu@MoS_2$	Cu	2.21 wt%	Hour	SR	*Applied Catalysis B: Environmental* 2019, 251, 87
Pd/MoS_2	Pd	1.0 wt%	Hour	SR	*Nat. Commun.*, 2018, **9**, 2120
Co-substituted Ru NSs	Co	6 atom%	Hour	SR	*Nat. Commun.* 2018, **9**, 4958
Rh_1/MoS_2	Rh	0.27 wt%	Hour	SR	*J. Am. Chem. Soc.* 2019, *10.1021/jacs.9b06628*
$Co\text{-}^sMoS_2$	Co	3 wt%	Hour	SR	*Chem. Sci.*, 2018, **9**, 4769
Co/NMC-LT900	Co	4.66 wt%	Hour	SR	*Nat. Commun.* 2019, **10**, 606
$Pt_{1.1}/BP_{defect}$	Pt	1.1 wt%	Hour	SR	*Angew. Chem.* 2019, **131**, 1175
Fe SAs/N-G	Fe	4.3 wt%	Hour	USM	*Adv.Mater.* 2019, 31, 1904496
RuAu SAAs	Au	15.35 atom%	Min	LAL	*Adv. Energy Mater.* 2019, **9**, 1803913
Fe^{3+}–N–C	Fe^{3+}	2.8 wt%	Hour	PM	*Science* 2019, **364**, 1091
Co SAs/N-C	Co	4 wt%	Hour	PM	*Angew. Chem. Int. Ed.* 2016, **55**, 10800
SA-Mo/NPC	Mo	9.54 wt%	Hour	PM	*Angew. Chem. Int. Ed.* 2019, **58**, 2321
CoN_x/C	Co	0.14 wt%	Hour	PM	*Nat. Commun.* 2015, **6**, 7992
Zn/CoN-C	Zn/Co	0.33 / 0.14 wt%	Hour	PM	*Angew. Chem. Int. Ed.* 2019, **58**, 1
W-SAC	W	1.21 wt%	Hour	PM	*Adv. Mater.* 2018, **30**, 1800396
Pt_1/N–C	Pt	0.43 wt%	Hour	PM	*Nat. Commun.* 2019, **10**, 3663
SA-Fe/NG	Fe	0.6 atom%	Hour	PM	*PNAS* 2018, **115**, 6626
$Mo_1N_1C_2$	Mo	1.32 wt%	Hour	PM	*Angew. Chem. Int. Ed.* 2017, **56**, 16086
Co–N–C@F127	Co	1.0 atom%	Hour	PM	*Energy Environ. Sci.* 2019, **12**, 250
Mn-N-C	Mn	3.03 wt%	Hour	PM	*Nat. Catal.* 2018, **1**, 935
Fe–NC SAC	Fe	12.1 wt%	Hour	PM	*Nat. Commun.* 2019, **10**, 1278
Co@MCM	Co	1.4 wt%	Hour	PM	*Energy Environ. Sci.* 2018, **11**, 1980
Co/NMC	Co	4.66 wt%	Hour	PM	*Nat. Commun.* 2019, **10**, 606

$Sn^{\delta+}$/NRGO	Sn	0.82 wt%	Hour	PM	*Adv. Mater.* 2019, **31**, 1808135
Fe-N-C	Fe	1.5 atom%	Hour	PM	*Energy Environ. Sci.* 2019, **12**, 2548
Ru@ZrO_2/NC	Ru	0.1 wt%	Hour	PM	*Chem* 2019, **5**, 1
Fe-N_xC_y	Fe	3.18 wt%	Hour	PM	*Nat. Commun.* 2019, **10**, 4290
Ir-N-C	Ir	0.2 wt%	Hour	PM	*Angew. Chem.* 2019, **131**, 9742
Fe-ZIF	Fe	0.5 atom%	Hour	PM	*J. Am. Chem. Soc.* 2017, **139**, 14143
Pd/Pt/Au-SAs	Pd/Pt/Au	0.16/0.41/0.18 wt%	min	PM	*Nat. Nanotechnol.* 2018, **13**, 856
Bi SAs/NC	Bi	0.2 wt%	Hour	PM	*J. Am. Chem. Soc.* 2019, **141**, 16569
SA M-GO	M=Ni, Co, Fe, Cu, Ag, Pd	2.8-7.9 wt%	Hour	PM	*Adv. Funct. Mater.* 2019, 1906157
M–NHGFs	Fe, Co, Ni	0.05 atom%	Hour	PM	*Nat. Catal.* 2018, **1**, 63
Cu-SA/N-C	Cu	0.54 wt%	Hour	DTBS	*Nat. Catal.* 2018, **1**, 781
Pt/Pd/Au SAs-DG	Pt/Pd/Au	2.1/2.6/1.8 wt%	Hour	DTBS	*J. Am. Chem. Soc.* 2019, **141**, 4505
Cu ISAS/NC	Cu	0.45 wt%	Hour	DTBS	*Nat. Commun.* 2019, **10**, 3734
A-Ni-C	Ni	1.5 wt%	Hour	ECM	*Nat. Commun.* 2016, **7**, 10667
SA Co-D 1T MoS_2	Co	3.54 wt%	Hour	ECM	*Nat. Commun.* 2019, **10**, 5231
PtSA-NT-NF	Pt	1.6 wt%	Hour	ERM	*Angew. Chem. Int. Ed.* 2017, **56**, 13694
SWNT/Pt	Pt	0.75 atom%	Hour	ERM	*ACS Catal.* 2017, **7**, 3121
$Mo_2TiC_2T_x$-Pt_{SA}	Pt	1.2 wt%	Hour	ERM	*Nat. Catal.* 2018, **1**, 985
Pt/np-$Co_{0.85}$Se	Pt	1.03 wt%	Hour	ERM	*Nat. Commun.* 2019, **10**, 1743
Ir_1/$Co(OH)_2$	Ir	1~2 wt%	min	ERM	*Nat. Commun.* 2020, **11**, 1215
M-MoS_2	M=Pt/Au/Pd	1.1/7/14 wt%	Hour	ERM	*Chem. Mater.* 2019, 31, 429
Fe/GD<br>Ni/GD	Fe/Ni	0.68 wt%<br>0.278 wt%	Hour	ERM	*Nat. Commun.* 2018, **9**, 1460
Pt/MgO	Pt	/	Hour	MSSL	*J. Am. Chem. Soc.* 1999, **121**, 3214
Pd/MgO	Pd	/	Hour	MSSL	*J. Am. Chem. Soc.* 2000, **122**, 3453
Pt/NGNs	Pt	2.1 wt%	Hour	ALD	*Nat. Commun.* 2016, **7**, 13638
Pd_1/graphene	Pd	0.25 wt%	Hour	ALD	*J. Am. Chem. Soc.* 2015, **137**, 10484
Pt/graphene	Pt	1.52 wt%	Hour	ALD	*Sci. Rep.* 2013, **3**, 1775
Pt/graphene	Pt	2.4 wt%	Hour	ALD	*Nat. Commun.* 2017, **8**, 1070
Pt_1/OLC	Pt	0.27 wt%	Hour	ALD	*Nature Energy* 2019, **4**, 512
Pt/La-Al_2O_3	Pt	1 wt%	Hour	HTATM	*Science* 2016, **353**, 6295
Pt/CeO_2	Pt	1 wt%	Hour	HTATM	*Science* 2017, **358**, 1419
FeN_4/GN	Fe	1.5 / 2.7 / 4.0 wt%	Hour	BMM	*Sci. Adv.* 2015, **1**, 1500462
MN_4/GN	M=Mn, Fe, Co, Ni, Cu	1.9 / 2.9 / 2.6 / 2.9 / 2.5 wt%	Hour	BMM	*Angew. Chem. Int. Ed.* 2016, **55**, 6708
Au/TiO_2	Au	~1 wt%	Hour	DP	*J. Am. Chem. Soc.* 2013, **135**, 3768

Au-SA/Def-TiO_2	Au	0.25 wt%	Hour	DP	*Adv. Mater.* 2018, **30**, 1705369
Pt/Cu(111)	Pt	1 wt%	Hour	EBD	*Science* 2012, **335**, 1209
Pt/α-MoC	Pt	2 wt%	Hour	TPC	*Nature* 2017, **544**, 80
Pt/KLTL	Pt	1 wt%	Hour	IE	*Angew. Chem.* 2014, **126**, 9050
Co-NG-MW	Co	1.1 wt%	Sec	MWH	*Adv. Mater.* 2018, **30**, 1802146
Pt-CA-CNF	Pt	0.24 wt%	Sec	MWH	*Nat. Nanotechnol.* 2019, **14**, 851
Ir SAs-rGA	Ir	14.8 wt%	Hour	OCM	*ACS Catal.* 2019, **9**, 9905
Ni-TpBpy	Ni	1.76 wt%	Hour	OCM	*J. Am. Chem. Soc.* 2019, **141**, 7615
(Rh) ZJU-28-1d	Rh^{+}	1~5.8 wt%	Hour	OCM	*J. Am. Chem. Soc.* 2013, **135**, 10586
$Mn^{II}T$	Mn^{2+}	0.013~0.14 wt%	Hour	OCM	*J. Am. Chem. Soc.* 2008, **130**, 4945
M/SiO_2	M=Cr^{3+}/Eu^{3+}/Y^{3+}	0.64 / 4.16 wt %/	Hour	OCM	*J. Am. Chem. Soc.* 2017, **139**, 8855
M/SiO_2	M=Cr^{3+}/Yb^{3+}/Eu^{3+}/Y^{3+}	0.91 / 3.97/ 2.20/ 2.20 wt %	Hour	OCM	*J. Am. Chem. Soc.* 2017, **139**, 8855
MOF-525-Co	Co^{2+}	6.01%	Hour	OCM	*Angew. Chem. Int. Ed.* 2016, **55**, 14310
MOF-525-Zn	Zn^{2+}	6.42%	Hour	OCM	*Angew. Chem. Int. Ed.* 2016, **55**, 14310

Note that the red rows represent the data of Pt-SAs/MoS_2 and others in this study.

Abbreviations: SSED, site-specific electrodeposition (this work); IWI, incipient wetness impregnation; PRM, photochemical reduction method; CP, co-precipitation; DP, deposition precipitation; TPC, temperature-programmed carburization; EBD, electric beam deposition; OCM, organometallic complex method; IE, ion exchange; MWH, microwave heating; SR, solution reduction; USM, ultrasound method; LAL, laser ablation in liquid; PM, pyrolysis method; DTBS, direct transformation of bulk materials to single atoms; ERM, electrochemical reduction method; ECM, electrochemical corrosion method; MSSL, mass-selected soft-landing method; ALD, atomic layer deposition; HTATM, high-temperature atom-trapping method; BMM, ball-milling method.

Supplementary Table 4. Optimization of model structures with different Pt adatom sites and the corresponding binding energies.

**Sample**	**Initial Pt position**	**Binding energy (eV)**	**Structural illustration of the Pt adatom site**
**Pt-SAs/MoS_2**	*Mo1/S1	-5.11	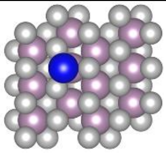
	Mo2	-4.52	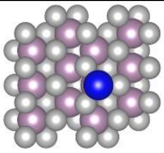
	S2	-4.12	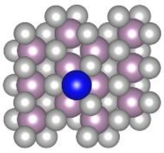
**Pt-SAs/WS_2**	*W1/S1	-5.18	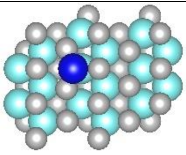
	W2	-4.57	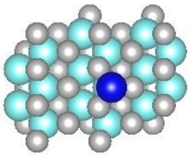
	S2	-4.25	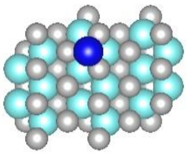
**Pt-SAs/$MoSe_2$**	*Mo1/Se2	-4.74	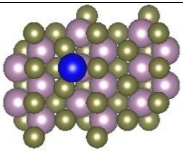
	Mo2	-4.16	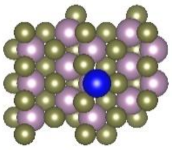
	Se1	-4.00	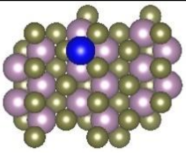

Atom key					
**Atom**	**Pt**	**Mo**	**W**	**S**	**Se**
**Representation**	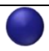	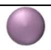	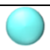	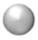	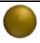

* The red rows represent the optimal structures and data for further DFT simulation. The initial position of Pt atoms at Mo1/S1 in Pt-SAs/MoS_2, W1/S1 in Pt-SAs/WS_2, and Mo1/Se2 in Pt-SAs/$MoSe_2$ were stabilized to the same structure after geometry optimization.

Supplementary Table 5. Fitting parameters of the Fourier transform of the first shell of the EXAFS spectra.

Sample	Scatter	CN	R (Å)	σ^2 (Å^2)	ΔE_0 (eV)	r factor
Pt-SAs/MoS_2	Pt–S	3.2	2.26	0.0051	2.11	0.0053
Pt foil	Pt–Pt	12.0	2.76	0.0040	8.20	0.0007

CN, coordination number; R, average bond distance; σ^2, Debye–Waller factor; ΔE_0, inner potential correction; r factor reflects the goodness of the fit (<2%).

Supplementary Table 6. Optimization of the model structures with different Cu adatom sites and the corresponding binding energies.

Sample	Initial Pt position	Binding energy (eV)	Illustration of Cu position
**Cu-SAs/MoS_2**	*Mo1	-3.45	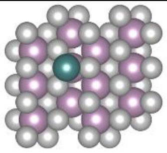
	Mo2	-3.10	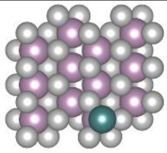
	S1	-3.20	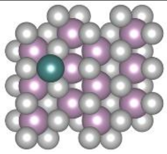
	S2	-3.09	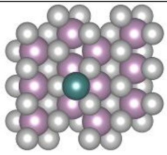
**Cu-SAs/WS_2**	*W1	-3.39	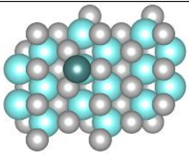
	W2	-3.02	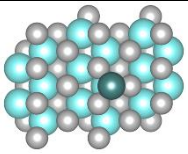
	S1	-3.10	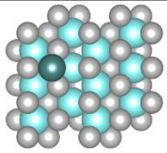
	S2	-3.02	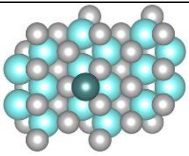
**Cu-SAs/$MoSe_2$**	*Mo1	-2.96	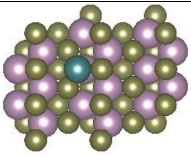

	Mo2	-2.58	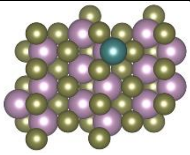
	Se1	-2.65	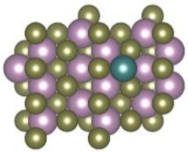
	Se2	-2.55	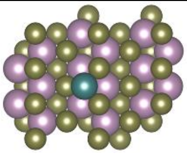
**Cu-SAs/WSe_2**	*W1	-2.15	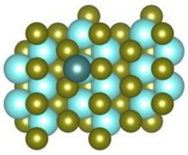
	W2	-1.80	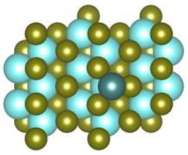
	Se1	-1.85	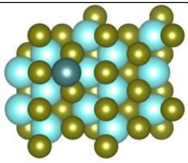
	Se2	-1.79	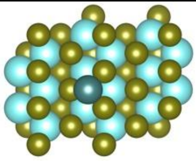

Atom key

**Atom**	**Cu**	**Mo**	**W**	**S**	**Se**
**Representation**	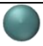	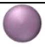	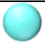	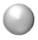	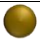

*The red rows represent the optimal structures and data for further DFT simulation.

Supplementary Table 7. Data (refer to Supplementary Fig. 15) for the underpotential shift ΔU_{p} and absolute values of binding energy ΔG_{BE} between a Cu atom and various TMD substrates obtained from DFT calculations.

Substrate	ΔU_{p} (V)	$\lvert\Delta G_{\mathrm{BE}}\rvert$ (eV)
MoS_2	0.197	3.45
WSe_2	0.102	2.15
$MoSe_2$	0.153	2.96
WS_2	0.185	3.39

Supplementary Table 8. The concentration of metal precursor for single-atom synthesis reported previously by various methods.

Catalyst	Metal Precursor	Concentration	Reference
Pt_1/N–C	PtTPP	1.27 mM	*Nat. Commun.* 2019, **10**, 3663
Pt-CA-CNF	H_2PtCl_6	0.5 mM	*Nat. Nanotechnol.* 2019, **14**, 851
Co-$^sMoS_2$	$Co(thiourea)_4^{2+}$	4 mM	*Nat. Chem.* 2017, **9**, 810
$Pt_{1.1}/BP_{defect}$	$Pt(acac)_2$	0.056 mM	*Angew. Chem.* 2019, **131**, 1175
Pt_1@Fe−N−C	$H_2PtCl_6·6H_2O$	9.65 mM	*Adv. Energy Mater.* 2018, **8**, 1701345
Pt-MoS_2	$H_2PtCl_6·6H_2O$	4.2 mM	*Energy Environ. Sci.* 2015, **8**, 1594
Ir-SACs	$Ir(acac)_3$	1.29 mM	*Angew. Chem.* 2019, **131**, 9742
Sn-SAs	$SnCl_2$	2.0 mM	*Adv. Mater.* 2019, **31**, 1808135
SA-Mo/NPC	$(NH_4)_6Mo_7O_{24}$	0.083 mM	*Angew. Chem. Int. Ed.* 2019, **58**, 2321
Pt/MoS_2	K_2PtCl_6	0.075 mM	*Nat. Nanotechnol.* 2018, **13**, 411
Pd1/ND@G	$Pd(NO_3)_2$	6 mM	*J. Am. Chem. Soc.* 2018, 140, 13142
Pd_1/TiO_2	H_2PdCl_4	0.25 mM	*Science* 2016, **352**, 797

Supplementary Table 9. Comparison of the HER activity of Pt-SAs/MoS_2 and others in this study with reported state-of-the-art single-atom catalysts in acid condition (electrolyte: 0.5 M H_2SO_4).

Catalyst	η (mV)	Tafel slope (mV dec^{-1})	η_{10} (mV)	TOF (s^{-1})	Reference
Pt-SAs/MoS_2	~0	31	59	175@200 mV	This work
Pt-SAs/$MoSe_2$	~0	28	67	163@200 mV	This work
Pt-SAs/WS_2	~0	28	32	273@200 mV	This work
Pd-SAs/MoS_2	~0	62	119	101@200 mV	This work
Pt_1/OLC	~0	36	38	90 @200 mV	*Nat. Energy* 2019, **4**, 512
A-Ni-C	~0	41	34	NA	*Nat. Commun.* 2016, **7**, 10667
Pt@PCM	~0	65.3	105	12@200 mV	*Sci. Adv.* 2018, **4**, 6657
A-Ni@DG	~0	31	70	45@200 mV	*Chem* 2018, **4**, 1
Pt/SWNT	~0	38	27	NA	*ACS Catal.* 2017, **7**, 3121
Pt_1@Fe-N-C	~0	42	60	NA	*Adv. Energy Mater.* 2018, **8**, 1701345
Pt/MoS_2	~0	96	145	NA	*Energy Environ. Sci.* 2015, **8**, 1594
Pt/hCNC	~0	24	15	7.67@20 mV	*Nat. Commun.* 2019, **10**, 1657
Co-$^{s}MoS_2$	~175	92	220	NA	*Chem. Sci.*, 2018, **9**, 4769
Co-NG	~30	82	147	1.189@200 mV	*Nat. Commun.* 2015, 6, 8668
Ni/graphene	~50	45	~180	0.8@300 mV	*Angew. Chem. Int. Ed.* 2015, **54**, 14031
Pt/NGNs	~0	29	~40	NA	*Nat. Commun.* 2016, **7**, 13638
Pd/MoS_2	~0	80	89	16.54@200 mV	*Nat. Commun.*, 2018, **9**, 2120
Ru SAs@PN	~0	38	24	4.29@50 mV	*Angew. Chem. Int. Ed.* 2018, **57**, 9495
mPF-Co-MoS_2	~55	74	156	NA	*Nat. Commun.* 2017, **8**, 14430
$Mo_2TiC_2T_x$-Pt_{SA}	~0	30	30	NA	*Nat. Catal.* 2018, **1**, 985
Ru_{SA}-N-S-$Ti_3C_2T_x$	~0	90	76	1.50@200 mV	*Adv. Mater.* 2019, 1903841
Pt-MoS_2	~85	104	210	NA	*Chem. Mater.* 2019, **31**, 429
Pt_1/MoO_{3-x}	~0	28.8	23.3	NA	*ChemCatChem* 2018, **10**, 946
Pt-GDY2	~0	46.6	65	NA	*Angew. Chem.* 2018, **130**, 9526
Co-NG-MW	~0	80	175	0.385@100 mV	*Adv. Mater.* 2018, **30**, 1802146
CoSAs/PTF	~0	50	94	NA	*J. Mater. Chem. A*, 2019, **7**, 1252
CoN_x/C	~0	57	133	6.5@200 mV	*Nat. Commun.* 2015, **6**, 7992
Pt_1/NPC	~0	28	25	100@100 mV	*ACS Catal.* 2018, **8**, 8450
W-SAC	~0	58	105	4.5@120 mV	*Adv. Mater.* 2018, **30**, 1800396
AC Pt-NG/C	~0	31	35	0.093@0 mV	*ACS Catal.* 2019, **9**, 8213
Pt_1/NMC	~0	26	30	NA	*Chem. Sci.* 2019, **10**, 2830
Pt/NiS@Al_2O_3	~0	35	34	NA	*J. Mater. Chem. A* 2018, **6**, 11783

Pt@MoS_2/NiS_2	~0	41	34	NA	*Small* 2018, **14**, 1800697
Fe/GD	~0	37.8	66	4.15@100 mV	*Nat. Commun*. 2018, **9**, 1460
Ni/GD	~0	45.8	88	1.59@100 mV	*Nat. Commun*. 2018, **9**, 1460
$MoS_{2-x}O_x$	~100	67	175	NA	*Nat. Commun*. 2018, **10**, 1246
Pd/Cu-Pt	~0	25	22.8	NA	*Angew. Chem. Int. Ed.* 2017, **56**, 16047
CoSAs/PTFs	21	50	94	NA	*J. Mater. Chem. A* 2019, **7**, 1252
PtSA-NT-NF	~0	NA	30	NA	*Angew. Chem. Int. Ed.* 2017, **56**, 13694
Cu@MoS_2	~50	51	131	70@200 mV	*Appl. Catal. B: Environ.* 2019, 251, 87
Pt SA/WO_{3-x}	~0	45	50	35@100 mV	*Angew.Chem.* 2019, 131, 16184
Ni_{SA}-MoS_2/CC	~0	74	110	NA	*Nano Energy* 2018, **53**, 458
Pt SAs/DG	~0	25	23	NA	*J. Am. Chem. Soc.* 2019, **141**, 4505
Pt-CeO_2	~0	35	~100	NA	*Electrochim. Acta* 2019, **297**, 155
MoS_2–Ni	53	81	161	NA	*Adv. Funct. Mater.* 2018, **28**, 1807086
PtSA/S–C	~0	47	53	NA	*Nat. Commun*. 2019, **10**, 4977
SA Co-MoS_2	~0	32	~60	7.82@100 mV	*Nat. Commun.* 2019, **10**, 5231

Note that the red rows represent the HER activity of Pt-SAs/MoS_2 and others in this study.

Supplementary Table 10. Comparison between the previously reported electrodeposition methods and SSED for ADMC synthesis.

Comparison	Traditional single-atom electrodeposition[16-20]	C, A-ED method reported by Zeng's group[21]	Our SSED method*
Loading amount	0.2~1.6 wt%	2.3 wt%	5.1 wt%
Time scale	>10 hours	minutes	minutes
Controlling deposition time	Need	Need	No need
Controlling metal precursor concentration	No need	Need	No need
Electrolyte	H_2SO_4	H_2SO_4/KOH containing metal precursor	H_2SO_4 containing metal precursor
Depositing site	Vacancies/edges/steps (into lattice)	Vacancies/edges/steps (into lattice)	Atop Mo (coordinating with three nearest neighbouring S)
Capability of adjusting electronic states of metals	No	Yes	No

*Note that the red column represents our site-specific electrodeposition method for single-atom synthesis.

Supplementary References

1 Petrii, O. A. Oviedo, O. A., Reinaudi, L., Garcia, S. G., Leiva, E. P. M.: Underpotential deposition. From fundamentals and theory to applications at the nanoscale. Series: monographs in electrochemistry. *J. Solid State Electrochem.* **20**, 2383-2385 (2016).

2 Huang, M. *et al.* In depth analysis of complex interfacial processes: in situ electrochemical characterization of deposition of atomic layers of Cu, Pb and Te on Pd electrodes. *RSC Advances* **2**, 10994-11006 (2012).

3 Gong, K., Su, D. & Adzic, R. R. Platinum-monolayer shell on $AuNi_{0.5}Fe$ nanoparticle core electrocatalyst with high activity and stability for the oxygen reduction reaction. *J. Am. Chem. Soc.* **132**, 14364-14366 (2010).

4 Sasaki, K. *et al.* Core-protected platinum monolayer shell high-stability electrocatalysts for fuel-cell cathodes. *Angew. Chem. Int. Ed.* **49**, 8602-8607 (2010).

5 Xing, Y. *et al.* Enhancing oxygen reduction reaction activity via Pd−Au alloy sublayer mediation of Pt monolayer electrocatalysts. *J. Phys. Chem. Lett.* **1**, 3238-3242 (2010).

6 Sasaki, K. *et al.* Highly stable Pt monolayer on PdAu nanoparticle electrocatalysts for the oxygen reduction reaction. *Nat. Commun.* **3**, 1115 (2012).

7 Gerischer, H., Kolb, D. M. & Przasnyski, M. Chemisorption of metal atoms on metal surfaces in correlation to work function differences. *Surf. Sci.* **43**, 662-666 (1974).

8 Kolb, D. M., Przasnyski, M. & Gerischer, H. Underpotential deposition of metals and work function differences. *J . Electroanal. Chem. Interfacial Electrochem.* **54**, 25-38 (1974).

9 Backes, C. *et al.* Functionalization of liquid-exfoliated two-dimensional 2H-MoS_2. *Angew. Chem. Int. Ed.* **54**, 2638-2642 (2015).

10 Wang, J., Wang, K., Wang, F. B. & Xia, X. H. Bioinspired copper catalyst effective for both reduction and evolution of oxygen. *Nat. Commun.* **5**, 5285 (2014).

11 Wang, Y. *et al.* Single-atomic Cu with multiple oxygen vacancies on ceria for electrocatalytic CO_2 reduction to CH_4. *ACS Catal.* **8**, 7113-7119 (2018).

12 Zou, X. & Zhang, Y. Noble metal-free hydrogen evolution catalysts for water splitting. *Chem. Soc. Rev.* **44**, 5148-5180 (2015).

13 Li, T., Liu, J., Song, Y. & Wang, F. Photochemical solid-phase synthesis of platinum single atoms on nitrogen-doped carbon with high loading as bifunctional catalysts for hydrogen evolution and oxygen reduction reactions. *ACS Catal.* **8**, 8450-8458 (2018).

14 Cheng, N. *et al.* Platinum single-atom and cluster catalysis of the hydrogen evolution reaction. *Nat. Commun.* **7**, 13638 (2016).

15 Li, Y. *et al.* MoS_2 nanoparticles grown on graphene: an advanced catalyst for the hydrogen evolution reaction. *J. Am. Chem. Soc.* **133**, 7296-7299 (2011).

16 Tavakkoli, M. *et al.* Electrochemical activation of single-walled carbon nanotubes with pseudo-atomic-scale platinum for the hydrogen evolution reaction. *ACS Catal.* **7**, 3121-3130 (2017).

17 Zhang, L., Han, L., Liu, H., Liu, X. & Luo, J. Potential-cycling synthesis of single platinum atoms for efficient hydrogen evolution in neutral media. *Angew. Chem. Int. Ed.* **56**, 13694-13698 (2017).

18 Xuan, N. *et al.* Single-atom electroplating on two dimensional materials. *Chem. Mater.* **31**, 429-435 (2019).

19 Jiang, K. *et al.* Single platinum atoms embedded in nanoporous cobalt selenide as electrocatalyst for accelerating hydrogen evolution reaction. *Nat. Commun.* **10**, 1743 (2019).

20 Zhang, J. *et al.* Single platinum atoms immobilized on an MXene as an efficient catalyst for the hydrogen evolution reaction. *Nat. Catal.* **1**, 985-992 (2018).

21 Zhang, Z. *et al.* Electrochemical deposition as a universal route for fabricating single-atom catalysts. *Nat. Commun.* **11**, 1215 (2020).

22 Knirsch, K. C. *et al.* Basal-plane functionalization of chemically exfoliated molybdenum disulfide by diazonium salts. *ACS Nano* **9**, 6018-6030 (2015).

23 Chou, S. S. *et al.* Ligand conjugation of chemically exfoliated MoS_2. *J. Am. Chem. Soc.* **135**, 4584-4587 (2013).

24 Eda, G. *et al.* Photoluminescence from chemically exfoliated MoS_2. *Nano Lett.* **11**, 5111-5116 (2011).

25 Liu, G. *et al.* MoS_2 monolayer catalyst doped with isolated Co atoms for the hydrodeoxygenation reaction. *Nat. Chem.* **9**, 810-816 (2017).

26 Paredes, J. I. *et al.* Impact of covalent functionalization on the aqueous processability, catalytic activity, and biocompatibility of chemically exfoliated MoS_2 nanosheets. *ACS Appl. Mater. Inter.* **8**, 27974-27986 (2016).

27 Voiry, D. *et al.* Covalent functionalization of monolayered transition metal dichalcogenides by phase engineering. *Nat. Chem.* **7**, 45 (2014).

28 Tang, Q. & Jiang, D. e. Mechanism of hydrogen evolution reaction on 1T-MoS_2 from first principles. *ACS Catal.* **6**, 4953-4961 (2016).

29 Voiry, D. *et al.* Conducting MoS_2 nanosheets as catalysts for hydrogen evolution reaction. *Nano Lett.* **13**, 6222-6227 (2013).

30 Radisavljevic, B., Radenovic, A., Brivio, J., Giacometti, V. & Kis, A. Single-layer MoS_2 transistors. *Nat. Nanotechnol.* **6**, 147 (2011).

31 Ghatak, S., Pal, A. N. & Ghosh, A. Nature of electronic states in atomically thin MoS_2 field-effect transistors. *ACS Nano* **5**, 7707-7712 (2011).

32 Yang, D., Sandoval, S. J., Divigalpitiya, W. M. R., Irwin, J. C. & Frindt, R. F. Structure of single-molecular-layer MoS_2. *Phys. Rev. B* **43**, 12053-12056 (1991).

33 Lukowski, M. A. *et al.* Enhanced hydrogen evolution catalysis from chemically exfoliated metallic MoS_2 nanosheets. *J. Am. Chem. Soc.* **135**, 10274-10277 (2013).

34 Yin, Y. *et al.* Contributions of phase, sulfur vacancies, and edges to the hydrogen evolution reaction catalytic activity of porous molybdenum disulfide nanosheets. *J. Am. Chem. Soc.* **138**, 7965-7972 (2016).

35 Qu, Y. *et al.* Direct transformation of bulk copper into copper single sites via emitting and trapping of atoms. *Nat. Catal.* **1**, 781-786 (2018).

36 Shi, Y. *et al.* Energy level engineering of MoS_2 by transition-metal doping for accelerating hydrogen evolution reaction. *J. Am. Chem. Soc.* **139**, 15479-15485 (2017).

37 Kwon, Y., Kim, T. Y., Kwon, G., Yi, J. & Lee, H. Selective activation of methane on single-atom catalyst of rhodium dispersed on zirconia for direct conversion. *J. Am. Chem. Soc.* **139**, 17694-17699 (2017).

38 Lang, R. *et al.* Hydroformylation of olefins by a rhodium single-atom catalyst with activity comparable to $RhCl(PPh_3)_3$. *Angew. Chem. Int. Ed.* **55**, 16054-16058 (2016).

39 Zhang, H. *et al.* Dynamic traction of lattice-confined platinum atoms into mesoporous carbon matrix for hydrogen evolution reaction. *Sci. Adv.* **4**, eaao6657 (2018).

40 Mahmood, J. *et al.* An efficient and pH-universal ruthenium-based catalyst for the hydrogen evolution reaction. *Nat. Nanotechnol.* **12**, 441 (2017).

41 Heising, J. & Kanatzidis, M. G. Exfoliated and Restacked MoS_2 and WS_2: Ionic or Neutral Species? Encapsulation and Ordering of Hard Electropositive Cations. *J. Am. Chem. Soc.* **121**, 11720-11732 (1999).

42 Golub, A. S., Zubavichus, Y. V., Slovokhotov, Y. L., Novikov, Y. N. & Danot, M. Layered compounds assembled from molybdenum disulfide single-layers and alkylammonium cations. *Solid State Ion.* **128**, 151-160 (2000).
